# In-hospital costs after severe traumatic brain injury: A systematic review and quality assessment

**DOI:** 10.1371/journal.pone.0216743

**Published:** 2019-05-09

**Authors:** Jeroen T. J. M. van Dijck, Mark D. Dijkman, Robbin H. Ophuis, Godard C. W. de Ruiter, Wilco C. Peul, Suzanne Polinder

**Affiliations:** 1 Department of Neurosurgery, Neurosurgical Center Holland, Leiden University Medical Center, Leiden, The Netherlands; 2 Department of Neurosurgery, Neurosurgical Center Holland, Haaglanden Medical Center, The Hague, The Netherlands; 3 Department of Neurosurgery, Neurosurgical Center Holland, Haga Teaching Hospital, The Hague, The Netherlands; 4 Department of Public Health, Erasmus Medical Center, Rotterdam, The Netherlands; Mayo Clinic, UNITED STATES

## Abstract

**Background:**

The in-hospital treatment of patients with traumatic brain injury (TBI) is considered to be expensive, especially in patients with severe TBI (s-TBI). To improve future treatment decision-making, resource allocation and research initiatives, this study reviewed the in-hospital costs for patients with s-TBI and the quality of study methodology.

**Methods:**

A systematic search was performed using the following databases: PubMed, MEDLINE, Embase, Web of Science, Cochrane library, CENTRAL, Emcare, PsychINFO, Academic Search Premier and Google Scholar. Articles published before August 2018 reporting in-hospital acute care costs for patients with s-TBI were included. Quality was assessed by using a 19-item checklist based on the CHEERS statement.

**Results:**

Twenty-five out of 2372 articles were included. In-hospital costs per patient were generally high and ranged from $2,130 to $401,808. Variation between study results was primarily caused by methodological heterogeneity and variable patient and treatment characteristics. The quality assessment showed variable study quality with a mean total score of 71% (range 48% - 96%). Especially items concerning cost data scored poorly (49%) because data source, cost calculation methodology and outcome reporting were regularly unmentioned or inadequately reported.

**Conclusions:**

Healthcare consumption and in-hospital costs for patients with s-TBI were high and varied widely between studies. Costs were primarily driven by the length of stay and surgical intervention and increased with higher TBI severity. However, drawing firm conclusions on the actual in-hospital costs of patients sustaining s-TBI was complicated due to variation and inadequate quality of the included studies. Future economic evaluations should focus on the long-term cost-effectiveness of treatment strategies and use guideline recommendations and common data elements to improve study quality.

## Introduction

Healthcare expenditures are rising worldwide and endanger the affordability of national healthcare systems. [1, 2] To secure their future existence, a thoughtful and righteous distribution of limited resources is essential. Policy makers and healthcare professionals are therefore increasingly expected to study the effectiveness of treatments and its associated costs. [3, 4] After all, the input from high quality cost research is required to make healthcare systems efficient and to achieve the highest quality of care for the lowest costs. [5]

Also in the field of traumatic brain injury (TBI), with an estimated total global annual burden of US$ 400 billion, research efforts are increasingly conducted towards cost- effectiveness. [6–10] After sustaining a TBI, in-hospital treatment is frequently required and generally associated with high costs. [11–14] In the USA, the 2010 TBI-related in-hospital charges totalled US$ 21.4 billion. [15] In-hospital costs after TBI are increasing annually and represent a substantial part of the total financial TBI burden. [15] The highest individual costs in TBI patients are generally seen in patients with severe TBI (s-TBI). [16] These patients also have the longest hospital or intensive care unit (ICU) length of stay (LOS) and the highest number of (neuro)surgical and medical interventions. [16–18] Despite their substantial healthcare consumption, these vulnerable patients show high rates of mortality and unfavourable outcome. Especially for these patients with poor outcome at high costs, a critical appraisal of treatment costs-effectiveness is essential to avoid ineffective expenditures and improve treatment decision-making. [19–22]

Two recent reviews on healthcare costs after TBI have reported about the considerable variation in healthcare costs after TBI between different studies and about the insufficient quality of the available cost studies.[7, 10] These reviews however were mainly focussed on the methodological quality of economic evaluations and therefore did not report the actual in-hospital costs. Insight into in-hospital costs and important components of the costs, such as healthcare utilization and other factors that drive these costs were not provided. This is important information for physicians and policymakers, because this information is needed for decision-making and for correct allocation of resources.

In this systematic review, we have therefore focussed on: (1) providing a detailed insight in the reported in-hospital costs for patients with s-TBI and (2) assessing the (quality of) study methodology.

## Methods

This systematic review was conducted according to the Preferred Reporting Items for Systematic Reviews and Meta-Analyses (PRISMA) guidelines. [23] (S1 Table) The study protocol was registered in the PROSPERO International Prospective Register of Systematic Review with registration number CRD42018081131.

### Literature search

A final systematic literature search was performed on the 8^th^ of August 2018 using the following databases: PubMed, MEDLINE, Embase, Web of Science, Cochrane library, CENTRAL, Emcare, PsychINFO, Academic Search Premier and Google Scholar. The search strategy was developed and conducted with the assistance of a trained clinical librarian. All relevant information on the literature search can be found in S1 Appendix. In addition to the search, the reference lists of all included articles were manually checked for additional relevant studies.

### Inclusion/Exclusion criteria

Studies were included when the in-hospital costs or in-hospital charges of a cohort of >10 patients with s-TBI were reported. Because the appellation “severe TBI” encompassed a range of brain injuries considered to be too varied for appropriate comparison the two most widely used classifications for s-TBI were applied: Glasgow Coma Scale (GCS) ≤8 and/or Abbreviated Injury Scale (AIS) ≥4. [24–26] We excluded reviews, commentaries, editorials, conference and meeting abstracts, unpublished data, non-English studies and studies that could not be found or retrieved in full text. Studies were also excluded when in-hospital costs related to acute care were not distinguishable from other costs like indirect non-healthcare related costs (e.g. loss of productivity), (in-hospital) rehabilitation or long-term costs. There were no restrictions on publication date or patient characteristics.

### Article selection and data extraction

First, duplicates, non-English and unretrievable records were excluded. Second, two reviewers (JD,MD) independently screened the titles and abstracts of the remaining studies and selected all potential eligible studies. Full-texts were independently reviewed by the same researchers and studies were included according to the above mentioned criteria. During the process, all disagreements were resolved through discussion until consensus or after consulting a third researcher (RO). Data extraction was performed in duplicate using pre-created data extraction sheets. Extracted data was then discussed and combined. Variables that were collected included: study details, study population, definition of TBI (including severity), healthcare consumption, details of costs research methodology and cost outcome results.

### Quality assessment

A 19-item checklist was used to assure an accurate quality assessment for the evaluation of in-hospital costs following s-TBI. The checklist was based on the CHEERS statement, which is developed to improve the reporting on economic evaluations. [27–30] We slightly adjusted the items from the CHEERS statement by specifying items like ‘target population and subgroups’ in clear definition of illness and TBI severity, because this was deemed necessary for proper interpretation of study results. Also we intentionally left out items like cost perspective, time horizon and discounting costs since these were considered not relevant for short term in-hospital costs. The final checklist covers items in the areas of study details, population, clinical data, cost data and study methodology. All relevant details can be found in S2 Appendix.

The quality assessment was independently performed by three reviewers (JD, MD, RO). Disagreements were reassessed and discussed in several meetings until consensus was reached. All items were scored according to a predefined scoring manual that included four options: yes (1), suboptimal (0.5), no (0) and not applicable (N/A). A double weight was assigned to several items that were considered to be particularly important in calculating and reporting in-hospital costs. Final scores represented study quality and were presented as a percentage of the maximum score per study. Scores per item and item category were also calculated. All items that were not applicable were excluded from score calculation. When studies used a statistical model, items were scored considering the clear use and description of the model input parameters and sources.

### Outcome

All relevant data was reported in a descriptive manner. In line with the inclusion criteria, patients were included from three different severity groups as they were reported in the included studies (GCS≤8, AIS≥4, AIS≥5). These subgroups were also used in the text and figures. In one figure, hospital LOS was presented by using black indicators (■) and ICU LOS by white indicators (□). A clear distinction between hospital costs and hospital charges, when known, was made by using black and white indicators respectively. In-text, both the reported hospital charges and hospital costs were presented as in-hospital costs. The Gross Domestic Product (GDP) per capita of the study country was included as reference value, to improve comparability between the reported costs. The reference year that was used, corresponded with the currency year. [31] All costs, including GDP per capita, were converted to US dollars (2015) using the CCEMG–EPPI-Centre Cost Converter. [32] This web-based tool utilizes Gross Domestic Product deflator index values and Purchasing Power Parities conversion rates provided by the International Monetary Fund. [33] In case a reference year was not provided we used the last year in which patients were included or, when unknown, the year of publication. Figures were designed with GraphPad Prism version 7.0.2.

## Results

### Literature search and study selection

**Fig 1:**
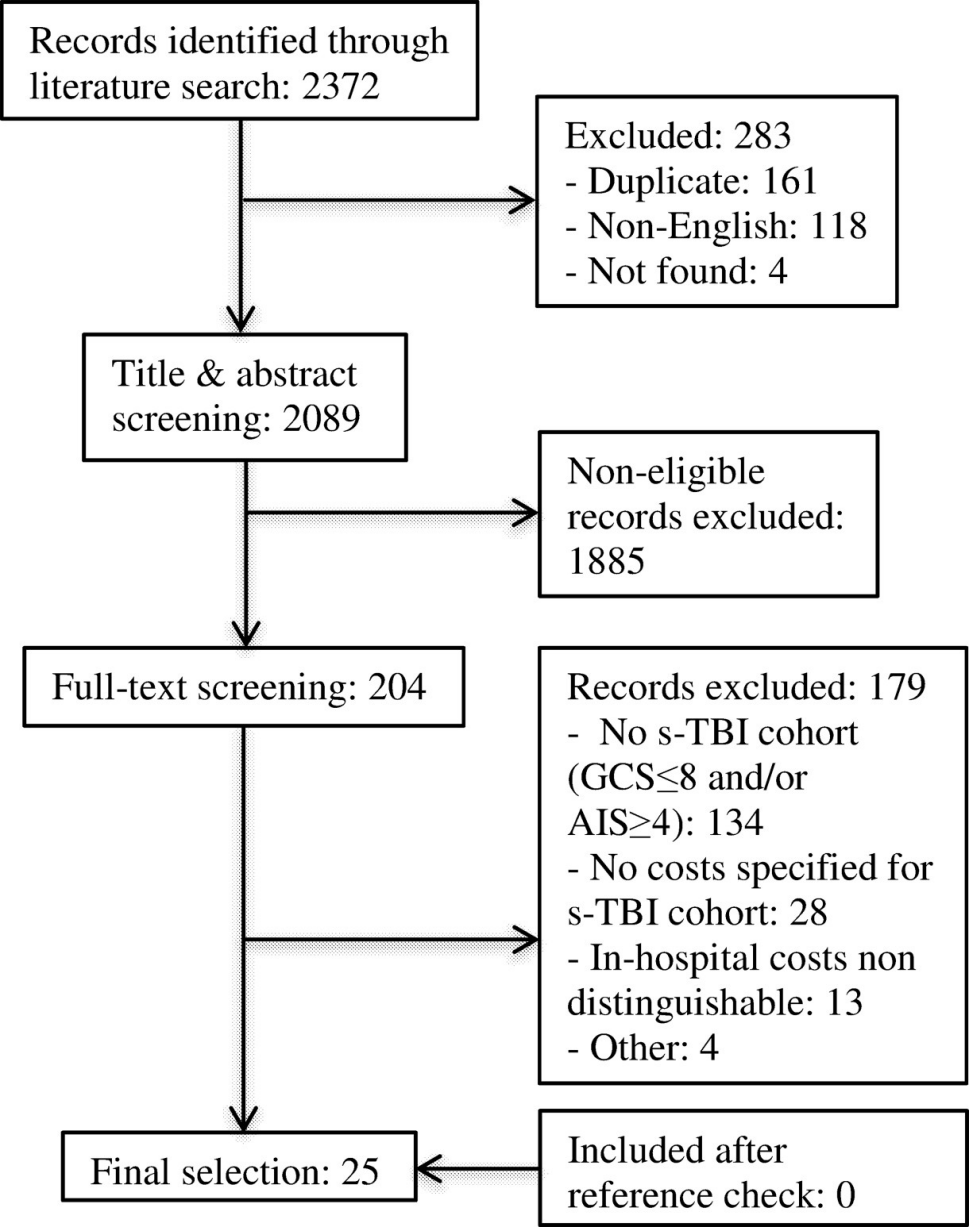
Flow chart of the article selection process.

The systematic literature search identified 2372 studies (Fig 1). First, a total of 283 duplicate, non-English or unfindable studies were removed. The remaining 2089 studies were screened on title and abstract, resulting in 204 studies considered eligible for full-text assessment. Studies were excluded because; (1) they did not include a s-TBI cohort defined by a GCS≤8 and/or AIS≥4 (N = 134), (2) they did not report hospital costs for patients with s-TBI (N = 28) or (3) in-hospital acute care costs were not distinguishable from other costs (N = 13). No additional studies were identified through the reference check. Ultimately, 25 articles were included in this systematic review.

### Study characteristics

The main study characteristics can be found in Table 1. Twelve studies were published after 2010, nine between 2000 and 2019, and four before 2000. Cohort size ranged from 20 to 7774 patients. [34, 35] Nineteen studies were conducted in high income countries of which sixteen in the USA. The majority of studies focused on adult patients, while some studies focused on paediatric [34, 36–38] and elderly patients. [35, 39] Nineteen studies (76%) had cost research in TBI patients as a research objective. TBI was often only defined by mentioning “TBI” or “head injury” (N = 9). Six studies provided only little additional information and nine studies used ICD (N = 8) and/or AIS codes (N = 2). Severity was defined by GCS (68%), by AIS (28%) or both (4%). The used GCS was obtained at admission (n = 7), the emergency department (n = 3) and the time remained unknown in 5 studies. A retrospective study design was used in 60% [35–37, 39–50], followed by a prospective design (16%) [34, 51–53] or a combination of both (12%). [54–56] Three studies used a statistical model. [38, 57, 58]

**Table 1:** Study details & results.

#	Study info^a^	Purpose	Study Design	Patient (N)	Definition of TBI	Severity definition	Cost data source	Details on cost calculation	Included costs	Currency (Y) / GDP per capita ^b^	Results ($ 2015) ^c^ *(% of GDP per capita)*
1	-Ahmed [40] -2007 -2002–2005 -USA	Evaluate the impact of early tracheostomy on s-TBI patients	Retrospective cohort study	55 s-TBI	TBI, not further specified	GCS≤8 at admission	Hospital accounting database	NP, most likely directly obtained from database	Total hospital charges	US$ (NP) / $52,876	ET (GCS 4.3±1.9): median $348,858 *(660%)* (95% CI: $293,682-$468,908) LT (GCS 4.5 ±1.8): median $396,917 *(751%)* (95% CI: $334,441-$520,808)
2	-Albrecht [39] -2017 -2008–2012 -USA	Provide charge estimates of TBI treatment for **elderly** patients	Retrospective cohort study	GCS<9:247 AIS4:688 AIS5:368	ICD-9-CM codes	GCS<9 at admission, AIS>3	Finance and billing department of (trauma) hospital and university	NP, most likely directly obtained from database	Hospital and physician charges. (Cost-to-charge ratio: 140.65%).	US$ (2012) / $53,681	GCS <9: $58,899 *(110%)* ± $74,194 AIS 4: $37,503 *(70%)* ± $58,025 AIS ≥5: $59,146 *(110%)* ± $87,230
3	--Andelic [57] --2014 -2005–2007 -Norway	Estimate long-term cost-effectiveness of rehabilitation trajectories	Decision-tree model	59 s-TBI	ICD-10 codes	GCS≤8 before intubation	Expected costs calculated from a reimbursement system using diagnosis related groups (DRG)	DRG reimbursement multiplied by the DRG cost weight for each patient	Total acute hospitalization costs for first 5 years post-injury	NOK (2009) / $87,894	All: $112,808 *(128%)* ± $68,327 Trajectory 1: $123,526 *(141%)* ± $50,911 Trajectory 2: $101,822 *(116%)* ± $81,725
4	-Brooks [41] -1995 -1989–1990 -USA	Determine the costs of health care services for TBI patients	Retrospective cohort study	28 s-TBI	TBI with AIS>0	AIS 4 and 5	Charges are obtained directly from all service providers	Services and billing records were added up to calculate actual/ estimated charges	Initial care charges including EMS, acute care charges and physicians charges of initial hospitalization	US$ (1993) / $40,211	Acute care: $123,303 *(307%)* Physicians: $25,767 *(64%)* Emergency Medical Services (EMS): $1,855 *(5%)*
5	-Bryant [42] -1993 -NP -USA	Find a high-quality cost- effective strategy for head injury rehabilitation	Retrospective cohort study	47 s-TBI	TBI, not further specified	GCS≤8 in ED	Costs are estimated from financial records of the health maintenance organization (HMO)	Unit costs are multiplied by utilized services	Acute medical care costs using actual operational costs.	US$ (NP) / $40,211	All: $24,205 *(60%)*
6	-Fakhry [43] -2004 -1991–2000 -USA	Determine effect of following BTF guidelines on outcome and charges	Cohort study with historical controls	830 s-TBI	TBI defined as blunt traumatic head injury with AIS-head > 2	GCS≤8	Trauma registry and individual chart review	NP, most likely directly obtained from registry of charts	Total charges (hospital room, critical care, nursing services, direct and indirect expenses, general hospital charges)	US$ (1997) / $44,428	1991–1994 (GCS 4.0): $51,634 *(116%)* 1995–1996 (GCS 3.5): $42,558 *(96%)* 1997–2000 (GCS 3.5): $40,002 *(90%)*
7	-Farhad [44] -2013 -1993-1994/ 2006–2007 -USA	Compare TBI-related hospitalization outcomes between 2 periods	Retrospective analysis of NIS data	317/ 288 s-TBI	ICD-9-CM codes	ICD/AIS 4–6	National Inpatient Sample (NIS) database (1993-1994/ 2006–2007)	NP, most likely directly obtained from database	Total charges of hospitalization	US$ (2006–2007) / $53,764	1993–1994: $21,427 ± $21,315 corrected for inflation: $29,999 *(56%)* 2006–2007: $65,002 *(121%)* ± $60,900
8	-Graves [36] -2016 -2007–2011 -USA	Evaluate guideline adherence on outcome and costs for **paediatric** s-TBI patients	Retrospective cohort study	235 s-TBI	ICD-9 codes, head AIS ≥ 3, history of trauma, abnormal admission head CT scan	GCS≤8 at admission	Total charged amounts most likely from hospitals, CCR from HCUP-KID or institution’s billing office	Obtained charges converted to costs with institution specific cost-charge ratio (CCR)	Total costs of hospitalization + ICU care	US$ (2012) / $53,681	Hospital mean: $106,969 *(199%)* (95% CI: $96,355 - $117,582) ICU mean: $84,843 *(156%)* (95%CI: $76,364 - $93,322)
9	-Ibrahim [51] -2007 -2003 --Malaysia	CEA of two neuro monitoring modalities in s-TBI management	Prospective observational CEA study	62 s-TBI	Severe head injury, traumatic in nature, not further specified	GCS≤8 and CT-scan features	All treatment costs measured using budget information	Macro and micro costing approach	Only direct provider costs calculated during admission	US$ (2002) / $5,379	Group 1 (GCS median 5.5, IQR 2.0): $10,356 ± $6,526 *(121%)* Group 2 (GCS median 6.0, IQR 2.0): $11,646 ± $8,168 *(152%)*
10	-Jaffe [34] -1993 -1987–1988 -USA	Assess acute and rehab costs of **paediatric** TBI patients	Prospective cohort study	20 s-TBI	Non-penetrating TBI with loss of consciousness	GCS≤8, at ED or before paralyzing agents	Hospital/physician charges from hospitals and physicians billing office	NP, most likely directly obtained from billing office	Charges used as proxy for costs. Initial acute care	US$ (1988) / $38,048	GCS3-8: $93,934 *(247%)* (range: $8,881–$328,857) AIS4: $32,375 *(85%)* ($16,378- $81,852) AIS5: $145,573 *(383%)* ($36,096-$328,857)
11	-Lehmkuhl [54] -1993 -1989–1992 -USA	Investigate factors that influence hospital charges for persons with TBI	Retrospective and prospective cohort study	111 s-TBI, 108 vs-TBI	TBI, defined as brain tissue damage caused by external force	GCS≤8, lowest score in first 24 hours	Copy of final billed charges submitted to designated payer	NP, most likely the submitted charges	Hospitalization costs (billed charges) for acute care excluding physicians fee	US$ (1989–1992) / $45,150	GCS6-8: $90,291 *(200%)* ± $72,243 GCS3-5: $141,813 *(314%)* ± $84,216
12	-Li [35] -2017 -2001–2007 -China	Epidemiological characteristics of **elderly** TBI patients	Retrospective analysis of Chinese Trauma Database data	5238 s-TBI 2536 c-TBI	ICD-9-CM codes	AIS4: severe AIS5-6: critical	Chinese Trauma Database dataset.	NP, most likely directly obtained from dataset	Hospitalization costs	US$ (NP) / $3,039	AIS4: $2,130 *(70%)* ± 3,881 AIS5-6: $3,586 *(118%)* ± 5,384
13	-Martini [45] -2009 -2004–2007 -USA	Resource utilization of brain tissue oxygen monitoring	Retrospective cohort study	629 s-TBI	TBI, not further specified	GCS≤8 at admission	Hospital administrative records	Charges converted to costs with institution specific CCR	Hospital costs	US$ (2007) / $54,204	Group 1 (GCS 5.6 ±2.3): $116,387 *(215%)* ± $85,034 Group 2 (GCS 5.1±2.2): $143,453 *(265%)* ± $88,079
14	-McGarry [46] -2002 -1997–1999 -USA	Examine treatment outcomes and costs of TBI	Retrospective analysis of database	2580 s-TBI 1147 c-TBI	ICD-9-CM codes	ICD/AIS4: severe ICD/AIS5: critical	Billed charges from a large multihospital database	Charges converted to costs with CCR	Hospitalization costs of acute treatment	US$ (1999) / $47,467	AIS4: $23,017 *(48%)* AIS5: $45,981 *(97%)*
15	-Morris [47] -2008 -2000–2005 -England/Wales	Investigate cost of care for hospitalised TBI patients	Retrospective analysis of database	2460 s-TBI 2573 c-TBI	TBI defined using 1998 AIS codes	AIS4: severe AIS5: critical	Trauma Audit and Research Network database and reference unit costs from different sources	Resource use from database and unit count multiplied by unit costs for other costs	National Health Service hospital costs	£ (NP) / $49,803	AIS4: $16,110 ± $30,088 *(60%)* AIS5: $29,504 ± $29,944 *(60%)*
16	-Palmer [55] -2001 -1994–1999 -USA	Report impact of TBI guideline implementation on outcome in s-TBI patients	Cohort study using retro- and prospective data	93 s-TBI	Closed head injury and evidence of brain injury on examination or CT-scan	GCS≤8 at admission	Patient records and/or financial data	NP, most likely directly obtained from records or financial data	Hospital charges	US$ (NP) / $47,467	Before implementation (GCS 6.4±0.7): $268,902 *(567%)* ± $31,761 After implementation (GCS 6.9±0.5): $401,808 *(846%)* ± $27,364
17	-Prang [48] -2012 -1995–2004 -Australia	Describe details of care services after transport related TBI	Analysis of a compensation database	316 s-TBI	Transport related-TBI, not further specified.	GCS3–8: severe	Accepted claims from Compensation Research Database	Mean costs calculated for each service category	Direct cost of healthcare over 5-year period post-injury	AUD $ (2009) / $46,885	Acute hospital services: $45,384 *(98%)* ± $38,720
18	-Salim [52] -2008 -2000–2004 -USA	Evaluate outcome of ARDS in patients with s-TBI	Prospectively collected cohort in ARDS dataset	28 s-TBI+ ARDS 56 s-TBI	Blunt trauma patients with TBI, AIS defined.	Head AIS ≥ 4	Hospital’s trauma registry	NP, most likely directly obtained from trauma registry	Hospital charges	US$ (NP) / $51,638	TBI+ARDS group (GCS 4±2): $258,790 *(501%)* ± $296,186 TBI group (GCS 5±2): $142,074 *(275%)* ± $198,248
19	-Schootman [49] -2003 -1996 -USA	Hospitalization charges for acute care in TBI patients in the USA	Population based descriptive study	1789 s-TBI	ICD-9-CM codes	ICD/AIS 4–6	National Inpatient Sample (NIS) of 1996	Database contains patient-level clinical and resource use information	Hospitalization billed charges for acute care	US$ (1996) / $43,035	Mean $47,004 *(109%)* ± $3,238; Median $20,886
20	-Siddiqui [56] -2015 -2002–2009 -Pakistan	Identify impact of early tracheostomy in s-TBI patients	Cohort study using retro- and prospective data	100 s-TBI	TBI, not further specified	GCS<8	Institution’s billing department	NP, most likely directly obtained from billing department	Inpatient treatment costs (ED, ICU, ward, lab, imaging, surgery)	US$ (2009) / $1,105	Group 1 (GCS 5.4±1.7): $8,811*(797%)* Group 2 (GCS 6.0±1.7): $10,934 *(990%)*
21	-White [37] -2001 -1991–1995 -USA	Determine predictors in **paediatric** s-TBI patients	Retrospective cohort study	136 s-TBI	Non-penetrating head injury, not further specified	GCS≤8 at admission to ED	NP: “were available”	Charges converted to costs using hospital based CCR	Hospitalization costs	US$ (1998) / $45,866	Survivors (GCS 5.4±1.9): $12,247 *(27%)* ($2,199-$127,555) Non-survivors (GCS 3.4±0.8): $7,081 *(15%)* ($2,305-$32,622)
22	-Whitmore [58] -2012 -N/A -USA	Determine the cost-effectiveness of treatment strategies in s-TBI patients	Decision-analytical model	N/A	TBI, not further specified	GCS≤8 and motor component of ≤5 at admission	Obtained from literature and Medicare reimbursement rates	Cost calculations follow general principles earlier described in literature and methods section	Direct acute medical care costs, primarily associated with the initial hospitalization	US$ (2011) / $52,910	Comfort care: GOS1: $60,582 *(115%)* GOS2-3: $111,067 *(210%)* GOS4-5: $43,753 *(83%)* Routine care: GOS 1: $77,410 *(146%)* GOS 2–3: $136,309 *(258%)* GOS4-5: $52,167 *(99%)* Aggressive care: GOS1-5: $124,725 *(236%)*
23	-You [50] -2018 -2015–2016 --Malaysia	Assign costs to treatment of surgically treated patients with TBI	Retrospective cohort study	26 s-TBI	ICD-10 codes	GCS3-8 on presentation	Hospital revenue department, finance department and financial reports	Micro- and macro- costing methods. Activity units multiplied by unit costs	Total cost of treatment (including hospitalization, surgery and investigations)	US$ (2016) / $9,416	GCS3-8: $8,964 *(95%)* ± $5,753
24	Yuan [53] -201) -2004 -China	Acute treatment costs for TBI	Prospective observational multicentre study	2500 s-TBI	TBI diagnosis was made by admitting neurosurgeons or ER physicians and confirmed by CT	GCS≤8 at admission	Unsubsidized total hospital billings	NP, most likely directly obtained from hospital billings	Total acute hospitalization treatment costs	US$ (2004) / $1,859	GCS3-8: median $3,115 *(168%)* ($1,468 - $6,046) Isolated TBI: $2,844 *(153%)* TBI with other injury: $3,207 *(173%)*
25	-Zapata-Vazquez [38] -2017 -N/A --Mexico	Cost-effectiveness of ICP monitoring in **paediatric** s-TBI patients	Decision-tree model	Based on 33 s-TBI patients	TBI, not further specified	GCS3-8	Most costs taken from official journal of the federation. Medicine price catalog, ICP probe price provided by supplier.	Amount of supplies multiplied by unit price	Costs of hospitalization (direct medical costs + clinical complications) medicines, laboratory, imaging, surgery, LOS ICU/Ward.	Mex$ (2015) / $9,291	ICP monitoring group (GCS 5.5±1.7): $66,263 *(713%)* ± $31,436 Control group (GCS 7.0±1.5): $41,783 *(450%)* ± $10,622

AIS, Abbreviated Injury Scale; ARDS, Adult Respiratory Distress Syndrome; BTF, Brain Trauma Foundation; CCR, Cost to Charge Ratio; CEA, Cost Effectiveness Analysis; CT, Computed Tomography; c-TBI, critical TBI; DRG, Diagnosis Related Groups; ED: Emergency Department; EMS, Emergency Medical Services; ET, Early Tracheostomy; GCS, Glasgow Coma Scale; HCUP-KID, Healthcare Cost and Utilization Project—Kids’ Inpatient Database; HMO, Health Maintenance Organization; ICD-10, International Classification of Diseases, 10^th^ Revision; ICD-9-CM, International Classification of Diseases, Ninth Revision; ICP, Intracranial Pressure; ICU, Intensive Care Unit; LOS, Length of Stay; LT, Late Tracheostomy; N/A, not applicable; N, Number; NIS, National Inpatient Sample; NP, Not provided; s-TBI, severe Traumatic Brain Injury; TBI, Traumatic Brain Injury; vs-TBI, very severe Traumatic Brain Injury; Y, Year

^a^ Name first author [reference #]—year of publication—Cohort inclusion period—Study country.

^b^ GDP per capita from year of currency and converted to $ 2015.

^c^ When available, severity defined by GCS was further specified by adding the mean GCS ± SD. (Unless stated otherwise)

### Quality of study methodology

The results of the quality assessment are presented in detail in S2 Table. Study quality was variable with an average total score of 71% and a range of 48% to 96%. Seven studies achieved a score above 80%, representing “high quality”. [36, 38, 39, 47, 50, 53, 58] Especially items in the ‘cost data’ subgroup scored poorly (49%). All but one study mentioned their cost data source, but a clear description was missing in 24%. Also, the design and methods of costs analysis were not mentioned in 36% and were unclear in another 16%. Eleven studies properly assessed hospital activity data but only three studies appropriately valued and reported unit costs. Hospital costs were disaggregated in 20% of studies and in 52% charges were reported instead of costs. Major assumptions were tested in a sensitivity analysis in only 16% and a reference year was missing in 14% of the studies. The subgroups ‘study details’, ‘population’ and ‘methodology’ had the highest scores (100%, 87% and 78%). There were infrequent statements on source of funding and conflicts of interest, unsatisfying TBI definitions and inadequate evaluation of study findings.

### Hospital costs & healthcare consumption

**Fig 2:**
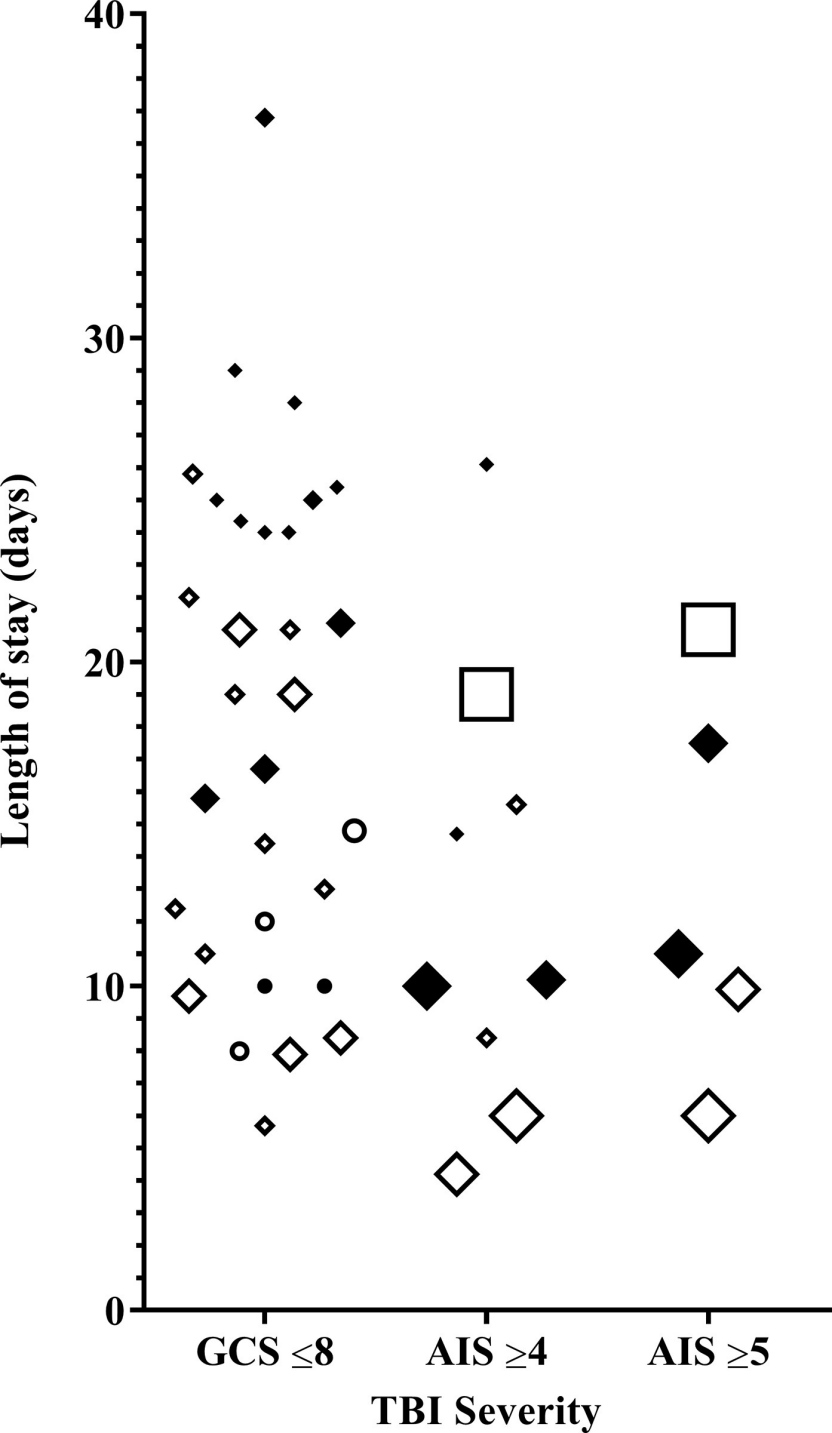
In-hospital costs and in-hospital charges of a patient with s-TBI. Black indicators represent in hospital costs, while white indicators represent in-hospital charges. A bigger indicator size, represents a bigger study cohort size. ● ○: Paediatric ♦ ◊: Adult ■ □: Elderly.

The median reported in-hospital costs per patient were $55,267 (mean $87,634) and ranged from $2,130 to $401,808 (Fig 2). The lowest costs were seen in studies from China, Pakistan and Malaysia ($2,130 to 10,356) [35, 50, 51, 53, 56] and in a subgroup of paediatric non-survivors in the USA ($7,081). [37] The highest in-hospital costs ($258,790 to $401,808) were found in three studies describing different patient cohorts from the USA. [40, 52, 55] The in-hospital costs as percentage of the GDP per capita (median 128%, mean 234%) were highly variable and ranged from 15% to 990%. [37, 56] Mean percentages were not significantly different between high and lower income countries and between charges and costs (204% vs. 333% and 289% vs. 202%).

**Fig 3:**
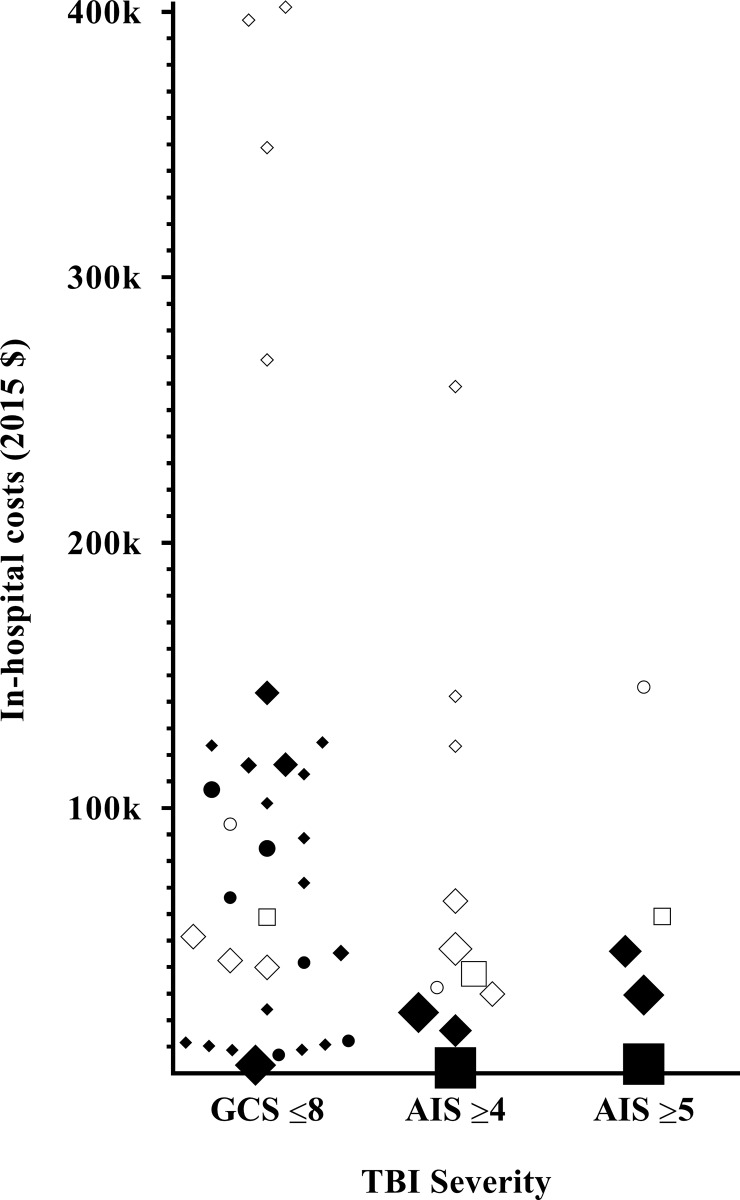
ICU and hospital length of stay of a patient with s-TBI. Black indicators represent hospital length of stay, while white indicators represent ICU length of stay. A bigger indicator size, represents a bigger study cohort size. ● ○: Paediatric ♦ ◊: Adult ■ □: Elderly.

Fourteen studies reported LOS for patients with s-TBI, also showing major variation (Fig 3). [35, 36, 38, 40, 43, 45–47, 50–52, 54–56] ICU LOS ranged from 7.9 to 25.8 days (GCS≤8) [40, 43], 6 to 19 days (AIS≥4) and 6 to 21 days (AIS≥5). [35, 47] Hospital LOS ranged from 10 to 36.8 days (GCS≤8) [38, 54], 10 to 26.1 days (AIS≥4) [47, 52] and 11 to 17.5 days (AIS≥5). [46, 47]

Some studies reported costs related to acute care to be 46% to 67% of total hospitalization costs, while inpatient rehabilitation costs accounted for 26% to 41%. [41, 42, 54, 57] Various studies found that costs related to hospital LOS and ICU LOS were the main drivers of hospital costs. [36, 38, 39, 47, 50, 53] Costs related to ICU care comprised the biggest part of total hospital costs (51–79%), followed by costs related to ward admission (12–38%), surgery (4–8%) and imaging/laboratory (<3%). [36, 38, 47] Physician charges were reported to be 12% to 20% of total costs. [39, 41] One study included the salary of paramedics and found salary to be the most important contributor (71–79%) to total provider costs. [39, 41, 51] The majority of costs, up to 90%, were made in the first year after trauma and were generally associated with TBI-related hospitalization costs. [41, 48, 57] The share of acute hospital services (18%) and rehabilitation (27%) on total costs decreased when a long-term follow-up period was used. [52]

Several studies provided some additional information on clinical factors that were associated with reported costs. A higher TBI severity was generally related with an increased LOS and costs. [34, 35, 37–39, 41, 42, 46–50, 53, 54] Even among patients with a s-TBI, patients with a GCS3-5 or AIS = 5 were more expensive than patients with a GCS6-8 or AIS = 4, respectively. [34, 35, 39, 40, 46, 47, 54] A higher overall injury severity was also related with higher costs. [39, 47, 53] Male gender was linked with higher costs [35, 39, 53] and two studies mentioned that a higher age was more expensive. [47, 50] Costs were also influenced by trauma mechanism and were higher for motor vehicle accidents and gunshot wounds and lower after an assault to the head. [34, 35, 39, 46, 53, 54] The use of surgical intervention, intracranial pressure monitoring or mannitol were all related to longer LOS and higher costs. [37, 38, 45, 53, 54] Also, the introduction of guidelines and evidence based medicine protocols appeared to increase LOS and hospital costs [43, 55], while improvement of guideline adherence did not change ICU and hospital costs in another study. [36] Three studies related costs to outcome and found lower costs for patients that died or made a good recovery. [37, 53, 58]

## Discussion

This systematic review demonstrates that the in-hospital costs related to acute care for patients with s-TBI are generally high and increase with severity of TBI and overall severity of the injury. Both healthcare consumption and in-hospital costs are highly variable between studies and associated with factors such as mechanism of injury and treatment strategy.

Three previous reviews on costs after TBI were generally in line with our results, but results were difficult to compare with the present review due to differences in study objectives and substantial variation between the included studies that was mainly caused by differing methodological and clinical characteristics. [7, 59, 60] Elaborating on these reviews, we specifically investigated the in-hospital costs related to acute care for patients with s-TBI aiming to reduce variation and improve study comparability. Methodological and clinical heterogeneity remained present, likely contributing to the variation in in-hospital costs between studies. The highest in-hospital costs were found in studies from the USA that reported charges instead of costs. Because hospital charges are not actual costs and usually higher than hospital costs, this increased total amounts. Charges are also often non-transparent and the resultant of deals between hospitals and insurance companies or other stakeholders. It is therefore preferred to calculate and report total costs by using healthcare utilization with its corresponding unit costs. Also, USA healthcare expenditures are twice as high as expenditures in other high-income countries. [2, 61] While healthcare utilization patterns were rather similar between high-income countries, the higher expenditures were especially caused by higher prices of labour, goods, pharmaceuticals and administrative costs. [2, 62] Large international differences were also seen between European countries when assessing injury related hospitalization costs. [63] Likewise, the lowest in-hospital costs were found in studies from lower-income countries, which is also in accordance with literature. [64] These absolute costs are lower because of lower prices, lower treatment intensity and higher mortality rates with associated lower resource utilization. [64, 65] In-hospital costs reported as percentage of GDP per capita were however not significantly different between high and low income countries, suggesting a similar financial impact for patients. Differences in costs might also be caused by hospital associated factors (e.g. level of trauma center, volume, treatment protocols) and by the major epidemiological differences of trauma populations between countries. [6] The different timeframes included in this review could also contribute to variation, since treatment strategies have changed over time and healthcare costs have been increasing globally over the years. [15, 64, 66] Comparing in-hospital costs from different healthcare systems in different timeframes is therefore problematic.

As in literature, the identified in-hospital costs increase with higher TBI severity. [9, 16, 60, 67] Costs increase because they primarily consist of costs related to LOS and surgical interventions and because the utilization of both is higher in more severely injured TBI patients. [68–71] After all, healthcare expenses are equal to utilization multiplied by associated prices. [62] Also in other studies, physician charges are another important contributor to in-hospital costs. [2, 72] Length of stay results and its variability seemed to be in accordance with literature, but were difficult to compare due to this variation. [68, 69] Like in previous research, extracranial injuries and overall injury severity contributed to higher healthcare consumption and in-hospital costs. [67, 69, 73–75] Distinguishing costs that are related to TBI or associated extracranial injuries is nearly impossible. Therefore, four studies explicitly investigated patients with isolated-TBI. [44, 51, 53, 56] Motor vehicle accidents and gunshot wounds were reported to be related to higher costs, most likely because of higher injury severity and accompanying extracranial injuries. Although a higher age is often considered to be more expensive, only few studies mentioned this and comparison between the age groups did not show obvious differences in LOS or in-hospital costs. [15, 63, 67, 73]

Hospital and acute care costs were reported to be important constituents of total costs followed by in-patient rehabilitation. However, the limitations of a short follow-up period have been recognized before. [7] Although the in-hospital costs are obviously an important part, post-discharge rehabilitation and other long term care costs are also major contributors to the total costs after TBI. [12] When including the enormous long-term or lifetime costs and the loss of productivity, the share of in-hospital costs on the total burden significantly decreases. [12, 14, 76] A long-term follow up period would provide a better overview for two reasons. First, the assessment of patient outcome will be more accurate, because health problems might persist, improve or deteriorate several years after trauma. [77, 78] Second, the cost analysis will be more comprehensive, since a changing health situation influences healthcare consumption and productivity for both patients and relatives. Therefore, especially for establishing the cost-effectiveness of treatments, a long-term follow-up should be included.

The identified most important reasons for (outcome) variation were probably all caused by different study objectives. Study objectives determined study methodology and consequently also the studied participants, interventions and outcome. Although most study objectives included costs research, the major differences between them likely caused the aforementioned methodological and clinical heterogeneity. Heterogeneity has earlier been reported for TBI cost studies and complicates study comparison and outcome interpretation. [7, 10, 59, 60] Heterogeneity is not limited to TBI cost research, but is very common in general TBI research and likewise complicates comparability, generalizability and interpretation of other studies. [79–82]

Study quality also influenced interpretation of study results, since poor methodological quality compromises quality and therefore value of data. Two recent reviews specifically assessed the methodological quality of TBI cost evaluation studies and identified important limitations regarding the adherence to the methodological principles of economic evaluations. [7, 10] More specifically, these limitations include not reporting all relevant costs on a long-term or lifetime horizon, not discounting future costs, not performing incremental analysis of cost-effectiveness and applying sensitivity analysis. Our quality assessment found variable and overall inadequate study quality. Only few studies were considered high quality and especially items concerning the calculation and reporting of costs scored poorly. Cost results were often provided without relevant context. A description of costs analysis methods, required to understand and interpret the results, was frequently missing. Studies also rarely calculated in-hospital costs by transparently multiplying healthcare consumption with associated unit costs. Almost no study reported the highly informative and important disaggregated costs. Even reference years were missing in several studies. Because several studies did not focus on reporting costs after TBI, they might have scored low on our quality assessment, despite appropriately investigating their specific study objectives.

### Strengths and limitations

This systematic review benefits from an extensive literature search in multiple databases and strict inclusion criteria, which improve study comparability and interpretation of results. The PRISMA guidelines were used during the review process and the quality assessment made use of a checklist that was based on the CHEERS statement and allowed the critical appraisal of the included articles. Although the assignment of scores is partly subjective, our experiences regarding the quality assessment using this checklist were positive. In addition, this is by our knowledge, the first detailed overview of in-hospital costs in patients with s-TBI.

This study also has several limitations. The article selection criteria resulted in the exclusion of some patients, that were severely injured but lacked the required severity classification. Also, regarding in-hospital costs, studies were excluded that not clearly distinguished acute care in-hospital costs from rehabilitation costs, indirect costs or other non in-hospital costs. Data from these patients could have contributed to our results, but the introduction of additional methodological and clinical heterogeneity would have compromised comparability and interpretation of study results. In addition, the used TBI severity criteria have their limitations. The GCS has been criticized for its general applicability although it shows adequate reliability in a recent review. [24, 83] A patient can be scored ‘false-low’ due to intubation and sedation overestimating injury severity, while the severity of patients who quickly deteriorate after admission will be underestimated. Also, a decreased GCS is not always caused by TBI and could also be caused by extracranial injury alone. [84] Last, patients could be at the lower or the higher end of the spectrum within the GCS 3–8 group. This could have substantial impact on study results, because severity is related to costs. Regarding AIS, the classification system changed over time and the 2005 version codes similar injuries with a lower severity score compared to the 1998 version. [85] Also, some researchers suggest using AIS≥5 as severe, instead of AIS≥4. [86] Despite this, using both criteria is very relevant because they are the most widely used criteria for s-TBI. [24] Limiting the selection to patients with s-TBI improves comparability, but fails to assess the financial burden caused by minor and/or moderate TBI. Although individual costs are lower for these injuries, the total burden on society is much higher because of their more frequent occurrence. [16] Although the distinction is clearly made throughout, including hospital charges and hospital costs may have compromised comparability of study results. Since both are frequently reported, it did however make a comprehensive review of in-hospital expenses possible and points out the difficulty of cost research. Last, the focus on in-hospital costs, dramatically underestimated the total financial burden caused by s-TBI. [12, 14, 76]

### Future research

Because a righteous and ethical distribution of limited healthcare resources is essential to secure the future existence of successful healthcare systems around the world, policymakers increasingly request high quality evidence regarding the cost effectiveness of treatments. [3] To improve the future quality of TBI cost research, investigators should equalize methodological and clinical heterogeneity by using specific methodological guidelines and common data elements. [27, 87] As seen in this systematic review, one of the biggest challenges in TBI cost research is heterogeneity. Checklists could be helpful, but the development of international guidelines on economical evaluations for TBI patients is preferred. Patient outcome should be investigated along with the financial burden of treatments. Therefore, cost-effectiveness analysis should be included in upcoming trials investigating TBI treatment strategies. Patients from all ages should be investigated because all are confronted with the consequences of TBI. Because TBI related consequences and associated costs are variable over time, economic evaluations should include a long-term or even lifetime horizon. [6] All associated costs adding to the total burden on society, like indirect costs and loss of productivity, should be included to accurately map expenditures. Also, health and financial implications for family and proxies deserve investigation. Last, the use of accurate cost calculation methods using exact healthcare consumption and cost price data could further improve the accuracy of cost calculations and thus outcome results. [88, 89]

## Conclusions

We conclude that healthcare consumption and in-hospital costs for patients with s-TBI are generally high. In-hospital costs mostly consist of costs related to LOS and surgical interventions. The major variation of study results is primarily caused by methodological and clinical heterogeneity. Study quality was variable but often inadequate and especially items considered important in calculation and reporting of in-hospital costs scored poorly. High quality future economic evaluations could guide physicians and policy-maker in improving clinical decision-making and resource allocation. Studies should therefore focus on the long-term cost-effectiveness of treatments and improve both study quality and equality by using guidelines and common data elements.

## Supporting information

S1 Appendix Literature search strategy. (DOCX)

S2 Appendix Quality assessment information. (DOCX)

S1 Table PRISMA 2009 checklist. N/A: Not applicable.
(DOC)

S2 Table Results of the quality assessment. * Item scores with double weight.
(DOCX)
