# Correction: In-hospital costs after severe traumatic brain injury: A systematic review and quality assessment

**DOI:** 10.1371/journal.pone.0219529

**Published:** 2019-07-05

**Authors:** Jeroen T. J. M. van Dijck, Mark D. Dijkman, Robbin H. Ophuis, Godard C. W. de Ruiter, Wilco C. Peul, Suzanne Polinder

The images for Figs 2 and 3 are incorrectly switched. The image that appears as Fig 2 should be Fig 3, and the image that appears as Fig 3 should be Fig 2. The figure captions appear in the correct order. Please see the complete, correct Figs 2 and 3 below.

**Fig 2:**
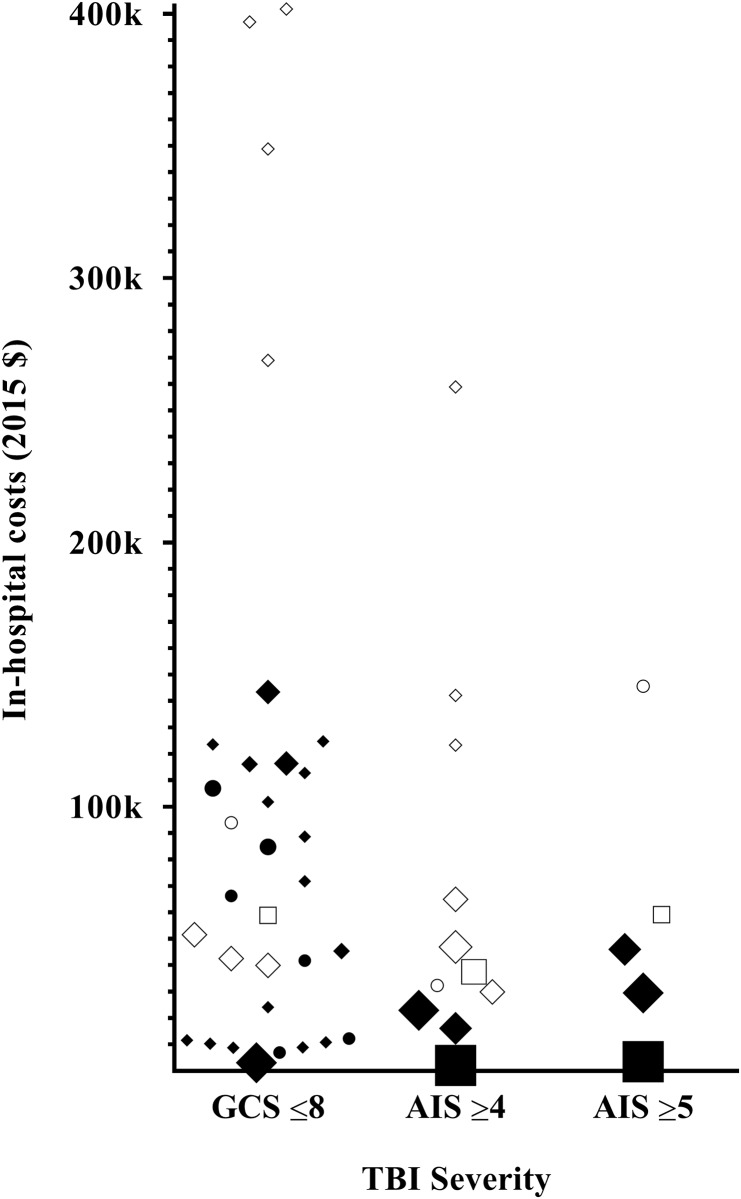
In-hospital costs and in-hospital charges of a patient with s-TBI. Black indicators represent in hospital costs, while white indicators represent in-hospital charges. A bigger indicator size, represents a bigger study cohort size. ● ○: Paediatric ♦ ◊: Adult ■ □: Elderly.

**Fig 3:**
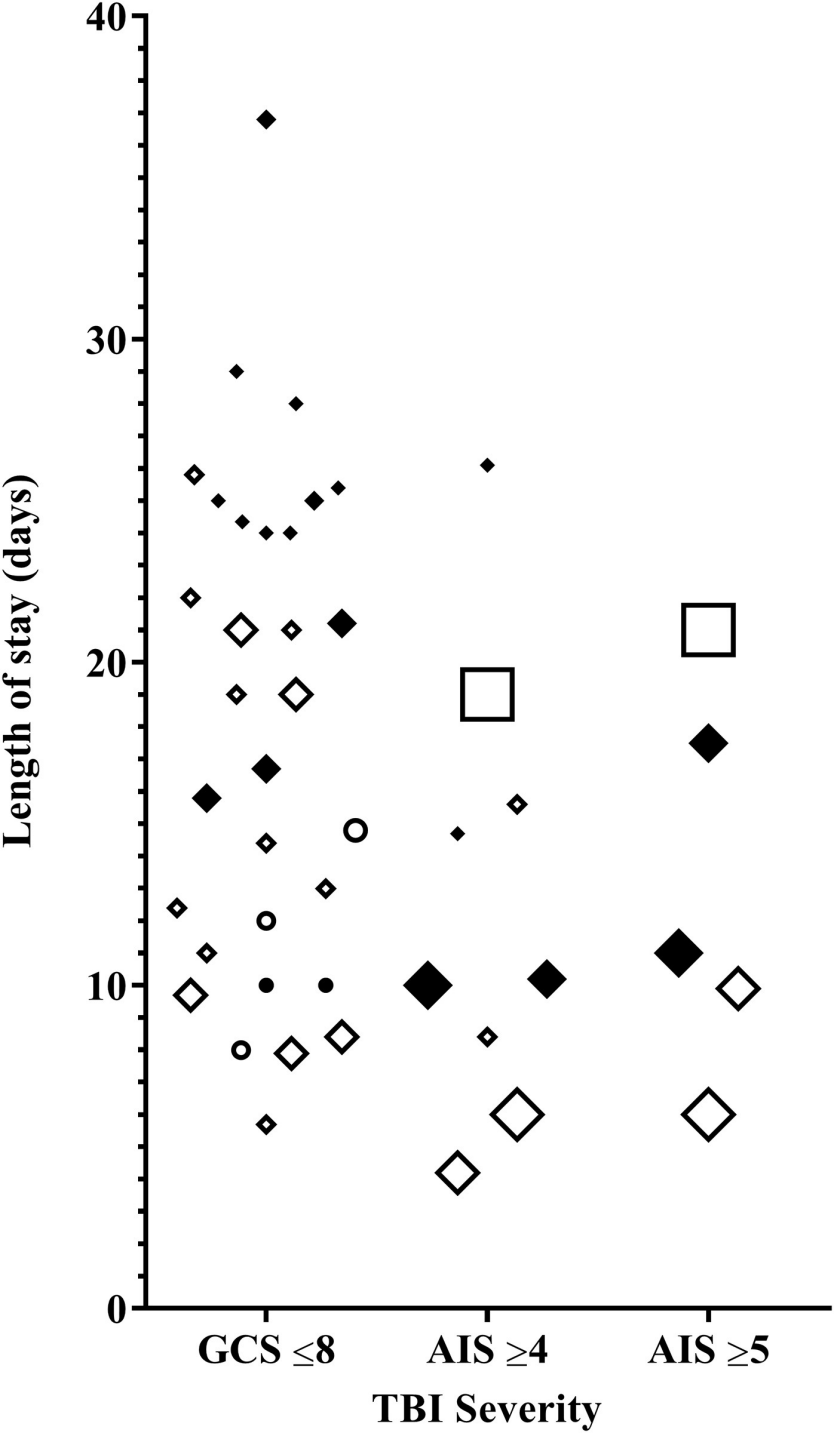
ICU and hospital length of stay of a patient with s-TBI. Black indicators represent hospital length of stay, while white indicators represent ICU length of stay. A bigger indicator size, represents a bigger study cohort size. ● ○: Paediatric ♦ ◊: Adult ■ □: Elderly.
