# Supplementary material for: In-hospital costs after severe traumatic brain injury: A systematic review and quality assessment
Source: PLoS One. 2019 May 9;14(5):e0216743. doi: 10.1371/journal.pone.0216743 (PMC6508680; doi:10.1371/journal.pone.0216743)
Supplement: S1 Appendix — (DOCX) [file pone.0216743.s001.docx]

**S1 Appendix. Literature Search Strategy**

Note 1: The systematic literature search took place on November 8^th^ 2017. When assembling the initial reference database (in Endnote X8), early identified double references were directly deleted (N=315). This resulted in 2126 records on which further assessment was performed. The systematic literature search was later updated (see note 2).

Note 2: A search update was performed on August 8^th^ 2018 and identical search strategies were used. All new articles (N=246) published from 2017 until August 8^th^ 2018 were added to the initially identified 2126 articles (N=2372) and further assessed. 111 duplicates were excluded. Then, 135 records were excluded after title & abstract assessment. Of 7 full-texts that were reviewed, one article was included.

In the article, the results of both searches were reported combined.

**PubMed (N=368)**- update (N=25), november 2017 – August 8th 2018

(**"Brain Injuries, Traumatic/economics"[Mesh] OR (("Economics"[majr] OR "Costs and Cost Analysis"[majr] OR "Cost of Illness"[majr] OR "cost"[ti] OR "costs"[ti] OR economic*[ti] OR financ*[ti] OR money*[ti] OR charg*[ti]) AND ("Brain Injuries, Traumatic"[mesh] OR "traumatic brain injury"[tw] OR "traumatic brain injuries"[tw] OR "traumatic brain injured"[tw] OR "traumatic brain hemorrhage"[tw] OR "traumatic brain hemorrhages"[tw] OR "traumatic brain haemorrhage"[tw] OR "traumatic brain damage"[tw] OR "traumatic brain stem injury"[tw] OR "traumatic brain"[tw] OR "brain trauma"[tw] OR "brain traumas"[tw] OR "**Traumatic Encephalopathy"[tw] OR "Traumatic Encephalopathies"[tw] OR "Brain Concussion"[tw] OR "Brain Concussions"[tw] OR "Commotio Cerebri"[tw] OR "Cerebral Concussion"[tw] OR "Cerebral Concussions"[tw] OR "Severe Concussion"[tw] OR "Severe Concussions"[tw] OR "Mild Concussion"[tw] OR "Mild Concussions"[tw] OR "Brain Contusion"[tw] OR "Brain Contusions"[tw] OR "**Contusio Cerebri"[tw] OR "Cerebellar Contusion"[tw] OR "Cerebellar Contusions"[tw] OR "Cerebral Contusion"[tw] OR "Cerebral Contusions"[tw] OR "Cortical Contusion"[tw] OR "Cortical Contusions"[tw])**))

**MEDLINE (N=378)**- update (N=46)

(exp **Brain Injuries, Traumatic/ec OR ((exp *"Economics"/ OR exp *"Costs and Cost Analysis"/ OR exp *"Cost of Illness"/ OR "cost".ti OR "costs".ti OR economic*.ti OR financ*.ti OR money*.ti OR charg*.ti) AND (exp "Brain Injuries, Traumatic"/ OR "traumatic brain injury".mp OR "traumatic brain injuries".mp OR "traumatic brain injured".mp OR "traumatic brain hemorrhage".mp OR "traumatic brain hemorrhages".mp OR "traumatic brain haemorrhage".mp OR "traumatic brain damage".mp OR "traumatic brain stem injury".mp OR "traumatic brain".mp OR "brain trauma".mp OR "brain traumas".mp OR "**Traumatic Encephalopathy".mp OR "Traumatic Encephalopathies".mp OR "Brain Concussion".mp OR "Brain Concussions".mp OR "Commotio Cerebri".mp OR "Cerebral Concussion".mp OR "Cerebral Concussions".mp OR "Severe Concussion".mp OR "Severe Concussions".mp OR "Mild Concussion".mp OR "Mild Concussions".mp OR "Brain Contusion".mp OR "Brain Contusions".mp OR "**Contusio Cerebri".mp OR "Cerebellar Contusion".mp OR "Cerebellar Contusions".mp OR "Cerebral Contusion".mp OR "Cerebral Contusions".mp OR "Cortical Contusion".mp OR "Cortical Contusions".mp)**))

**Embase (N=430)**- update (N=66)

(**(exp *"economic aspect"/ OR exp *"Cost"/ OR exp *"health care cost"/ OR "cost".ti OR "costs".ti OR economic*.ti OR financ*.ti OR money*.ti OR charg*.ti) AND ("traumatic brain injury"/ OR "traumatic brain injury".mp OR "traumatic brain injuries".mp OR "traumatic brain injured".mp OR "traumatic brain hemorrhage".mp OR "traumatic brain hemorrhages".mp OR "traumatic brain haemorrhage".mp OR "traumatic brain damage".mp OR "traumatic brain stem injury".mp OR "traumatic brain".mp OR "brain trauma".mp OR "brain traumas".mp OR "**Traumatic Encephalopathy".mp OR "Traumatic Encephalopathies".mp OR "Brain Concussion".mp OR "Brain Concussions".mp OR "Commotio Cerebri".mp OR "Cerebral Concussion".mp OR "Cerebral Concussions".mp OR "Severe Concussion".mp OR "Severe Concussions".mp OR "Mild Concussion".mp OR "Mild Concussions".mp OR "Brain Contusion".mp OR "Brain Contusions".mp OR "**Contusio Cerebri".mp OR "Cerebellar Contusion".mp OR "Cerebellar Contusions".mp OR "Cerebral Contusion".mp OR "Cerebral Contusions".mp OR "Cortical Contusion".mp OR "Cortical Contusions".mp)**) NOT conference review.pt

**Web of Science (N=205)**- update (N=32)

(ti=**("Cost" OR "health care cost" OR "cost" OR "costs" OR economic* OR financ* OR money* OR charg*) AND ts=("traumatic brain injury" OR "traumatic brain injury" OR "traumatic brain injuries" OR "traumatic brain injured" OR "traumatic brain hemorrhage" OR "traumatic brain hemorrhages" OR "traumatic brain haemorrhage" OR "traumatic brain damage" OR "traumatic brain stem injury" OR "traumatic brain" OR "brain trauma" OR "brain traumas" OR "**Traumatic Encephalopathy" OR "Traumatic Encephalopathies" OR "Brain Concussion" OR "Brain Concussions" OR "Commotio Cerebri" OR "Cerebral Concussion" OR "Cerebral Concussions" OR "Severe Concussion" OR "Severe Concussions" OR "Mild Concussion" OR "Mild Concussions" OR "Brain Contusion" OR "Brain Contusions" OR "**Contusio Cerebri" OR "Cerebellar Contusion" OR "Cerebellar Contusions" OR "Cerebral Contusion" OR "Cerebral Contusions" OR "Cortical Contusion" OR "Cortical Contusions")**)

**Cochrane Library (N=138)**- update (N=4)

**("traumatic brain injury" OR "traumatic brain injury" OR "traumatic brain injuries" OR "traumatic brain injured" OR "traumatic brain hemorrhage" OR "traumatic brain hemorrhages" OR "traumatic brain haemorrhage" OR "traumatic brain damage" OR "traumatic brain stem injury" OR "traumatic brain" OR "brain trauma" OR "brain traumas" OR "**Traumatic Encephalopathy" OR "Traumatic Encephalopathies" OR "Brain Concussion" OR "Brain Concussions" OR "Commotio Cerebri" OR "Cerebral Concussion" OR "Cerebral Concussions" OR "Severe Concussion" OR "Severe Concussions" OR "Mild Concussion" OR "Mild Concussions" OR "Brain Contusion" OR "Brain Contusions" OR "**Contusio Cerebri" OR "Cerebellar Contusion" OR "Cerebellar Contusions" OR "Cerebral Contusion" OR "Cerebral Contusions" OR "Cortical Contusion" OR "Cortical Contusions")**

**Emcare (N=228)**- update (N=25)

**((exp *"economic aspect"/ OR exp *"Cost"/ OR exp *"health care cost"/ OR "cost".ti OR "costs".ti OR economic*.ti OR financ*.ti OR money*.ti OR charg*.ti) AND ("traumatic brain injury"/ OR "traumatic brain injury".mp OR "traumatic brain injuries".mp OR "traumatic brain injured".mp OR "traumatic brain hemorrhage".mp OR "traumatic brain hemorrhages".mp OR "traumatic brain haemorrhage".mp OR "traumatic brain damage".mp OR "traumatic brain stem injury".mp OR "traumatic brain".mp OR "brain trauma".mp OR "brain traumas".mp OR "**Traumatic Encephalopathy".mp OR "Traumatic Encephalopathies".mp OR "Brain Concussion".mp OR "Brain Concussions".mp OR "Commotio Cerebri".mp OR "Cerebral Concussion".mp OR "Cerebral Concussions".mp OR "Severe Concussion".mp OR "Severe Concussions".mp OR "Mild Concussion".mp OR "Mild Concussions".mp OR "Brain Contusion".mp OR "Brain Contusions".mp OR **"Contusio Cerebri".mp OR "Cerebellar Contusion".mp OR "Cerebellar Contusions".mp OR "Cerebral Contusion".mp OR "Cerebral Contusions".mp OR "Cortical Contusion".mp OR "Cortical Contusions".mp))** NOT conference review.pt

**PsychINFO (N=270)**- update (N=13)

((DE "Costs and Cost Analysis" OR DE "Budgets" OR DE "Health Care Costs" OR DE "Budgets" OR DE "Health Care Costs" OR DE "Cost Containment" OR DE "Economics" OR DE "Behavioral Economics" OR DE "Evolutionary Economics" OR DE "Health Care Economics" OR DE "Neuroeconomics" OR DE "Pharmacoeconomics" OR DE "Economy" OR DE "Emerging Economies" OR DE "Knowledge Economy" OR DE "Finance" OR DE "Funding" OR DE "Health Care Economics" OR DE "Money" OR DE "Professional Fees" OR DE "Resource Allocation" OR TI(**"Cost" OR "health care cost" OR "cost" OR "costs" OR economic* OR financ* OR money* OR charg*)** OR SU(**"Cost" OR "health care cost" OR "cost" OR "costs" OR economic* OR financ* OR money* OR charg*)** OR MJ(**"Cost" OR "health care cost" OR "cost" OR "costs" OR economic* OR financ* OR money* OR charg*)** OR MA(**"Cost" OR "health care cost" OR "cost" OR "costs" OR economic* OR financ* OR money* OR charg***)) AND **(DE "Traumatic Brain Injury" OR DE "Brain Concussion" OR TI("traumatic brain injury" OR "traumatic brain injury" OR "traumatic brain injuries" OR "traumatic brain injured" OR "traumatic brain hemorrhage" OR "traumatic brain hemorrhages" OR "traumatic brain haemorrhage" OR "traumatic brain damage" OR "traumatic brain stem injury" OR "traumatic brain" OR "brain trauma" OR "brain traumas" OR "**Traumatic Encephalopathy" OR "Traumatic Encephalopathies" OR "Brain Concussion" OR "Brain Concussions" OR "Commotio Cerebri" OR "Cerebral Concussion" OR "Cerebral Concussions" OR "Severe Concussion" OR "Severe Concussions" OR "Mild Concussion" OR "Mild Concussions" OR "Brain Contusion" OR "Brain Contusions" OR "**Contusio Cerebri" OR "Cerebellar Contusion" OR "Cerebellar Contusions" OR "Cerebral Contusion" OR "Cerebral Contusions" OR "Cortical Contusion" OR "Cortical Contusions")** OR **SU("traumatic brain injury" OR "traumatic brain injury" OR "traumatic brain injuries" OR "traumatic brain injured" OR "traumatic brain hemorrhage" OR "traumatic brain hemorrhages" OR "traumatic brain haemorrhage" OR "traumatic brain damage" OR "traumatic brain stem injury" OR "traumatic brain" OR "brain trauma" OR "brain traumas" OR "**Traumatic Encephalopathy" OR "Traumatic Encephalopathies" OR "Brain Concussion" OR "Brain Concussions" OR "Commotio Cerebri" OR "Cerebral Concussion" OR "Cerebral Concussions" OR "Severe Concussion" OR "Severe Concussions" OR "Mild Concussion" OR "Mild Concussions" OR "Brain Contusion" OR "Brain Contusions" OR "**Contusio Cerebri" OR "Cerebellar Contusion" OR "Cerebellar Contusions" OR "Cerebral Contusion" OR "Cerebral Contusions" OR "Cortical Contusion" OR "Cortical Contusions")** OR **MJ("traumatic brain injury" OR "traumatic brain injury" OR "traumatic brain injuries" OR "traumatic brain injured" OR "traumatic brain hemorrhage" OR "traumatic brain hemorrhages" OR "traumatic brain haemorrhage" OR "traumatic brain damage" OR "traumatic brain stem injury" OR "traumatic brain" OR "brain trauma" OR "brain traumas" OR "**Traumatic Encephalopathy" OR "Traumatic Encephalopathies" OR "Brain Concussion" OR "Brain Concussions" OR "Commotio Cerebri" OR "Cerebral Concussion" OR "Cerebral Concussions" OR "Severe Concussion" OR "Severe Concussions" OR "Mild Concussion" OR "Mild Concussions" OR "Brain Contusion" OR "Brain Contusions" OR "**Contusio Cerebri" OR "Cerebellar Contusion" OR "Cerebellar Contusions" OR "Cerebral Contusion" OR "Cerebral Contusions" OR "Cortical Contusion" OR "Cortical Contusions")** OR **MA("traumatic brain injury" OR "traumatic brain injury" OR "traumatic brain injuries" OR "traumatic brain injured" OR "traumatic brain hemorrhage" OR "traumatic brain hemorrhages" OR "traumatic brain haemorrhage" OR "traumatic brain damage" OR "traumatic brain stem injury" OR "traumatic brain" OR "brain trauma" OR "brain traumas" OR "**Traumatic Encephalopathy" OR "Traumatic Encephalopathies" OR "Brain Concussion" OR "Brain Concussions" OR "Commotio Cerebri" OR "Cerebral Concussion" OR "Cerebral Concussions" OR "Severe Concussion" OR "Severe Concussions" OR "Mild Concussion" OR "Mild Concussions" OR "Brain Contusion" OR "Brain Contusions" OR "**Contusio Cerebri" OR "Cerebellar Contusion" OR "Cerebellar Contusions" OR "Cerebral Contusion" OR "Cerebral Contusions" OR "Cortical Contusion" OR "Cortical Contusions")))**

**CENTRAL (N=90)**- update (N=9)

**(("Cost" OR "health care cost" OR "cost" OR "costs" OR economic* OR financ* OR money* OR charg*) AND ("traumatic brain injury" OR "traumatic brain injury" OR "traumatic brain injuries" OR "traumatic brain injured" OR "traumatic brain hemorrhage" OR "traumatic brain hemorrhages" OR "traumatic brain haemorrhage" OR "traumatic brain damage" OR "traumatic brain stem injury" OR "traumatic brain" OR "brain trauma" OR "brain traumas" OR "**Traumatic Encephalopathy" OR "Traumatic Encephalopathies" OR "Brain Concussion" OR "Brain Concussions" OR "Commotio Cerebri" OR "Cerebral Concussion" OR "Cerebral Concussions" OR "Severe Concussion" OR "Severe Concussions" OR "Mild Concussion" OR "Mild Concussions" OR "Brain Contusion" OR "Brain Contusions" OR "**Contusio Cerebri" OR "Cerebellar Contusion" OR "Cerebellar Contusions" OR "Cerebral Contusion" OR "Cerebral Contusions" OR "Cortical Contusion" OR "Cortical Contusions"))**

**Academic Search Premier (N=99)**- update (N=23)

((TI(**"Cost" OR "health care cost" OR "cost" OR "costs" OR economic* OR financ* OR money* OR charg*)** OR KW(**"Cost" OR "health care cost" OR "cost" OR "costs" OR economic* OR financ* OR money* OR charg***)) AND **(TI("traumatic brain injury" OR "traumatic brain injury" OR "traumatic brain injuries" OR "traumatic brain injured" OR "traumatic brain hemorrhage" OR "traumatic brain hemorrhages" OR "traumatic brain haemorrhage" OR "traumatic brain damage" OR "traumatic brain stem injury" OR "traumatic brain" OR "brain trauma" OR "brain traumas" OR "**Traumatic Encephalopathy" OR "Traumatic Encephalopathies" OR "Brain Concussion" OR "Brain Concussions" OR "Commotio Cerebri" OR "Cerebral Concussion" OR "Cerebral Concussions" OR "Severe Concussion" OR "Severe Concussions" OR "Mild Concussion" OR "Mild Concussions" OR "Brain Contusion" OR "Brain Contusions" OR "**Contusio Cerebri" OR "Cerebellar Contusion" OR "Cerebellar Contusions" OR "Cerebral Contusion" OR "Cerebral Contusions" OR "Cortical Contusion" OR "Cortical Contusions")** OR **KW("traumatic brain injury" OR "traumatic brain injury" OR "traumatic brain injuries" OR "traumatic brain injured" OR "traumatic brain hemorrhage" OR "traumatic brain hemorrhages" OR "traumatic brain haemorrhage" OR "traumatic brain damage" OR "traumatic brain stem injury" OR "traumatic brain" OR "brain trauma" OR "brain traumas" OR "**Traumatic Encephalopathy" OR "Traumatic Encephalopathies" OR "Brain Concussion" OR "Brain Concussions" OR "Commotio Cerebri" OR "Cerebral Concussion" OR "Cerebral Concussions" OR "Severe Concussion" OR "Severe Concussions" OR "Mild Concussion" OR "Mild Concussions" OR "Brain Contusion" OR "Brain Contusions" OR "**Contusio Cerebri" OR "Cerebellar Contusion" OR "Cerebellar Contusions" OR "Cerebral Contusion" OR "Cerebral Contusions" OR "Cortical**

**Contusion" OR "Cortical Contusions"))**

**Google Scholar (N=235)**- update (N=3 non duplicates)

**allintitle: "cost"|"costs"|"economic"|"economics"|"finance" "traumatic brain injury"|"traumatic brain injuries"
allintitle: "money"|"charge"|"charges"|"charging"|"finances" "traumatic brain injury"|"traumatic brain injuries"
allintitle: "cost"|"costs"|"economic"|"economics"|"finance" "brain trauma"|"concussion"|"traumatic brain"
allintitle: "money"|"charge"|"charges"|"charging"|"finances" "brain trauma"|"concussion"|"traumatic brain"**

**Reasons for exclusion**

There were four main reasons for exclusion:
1: s-TBI cohort has not been not defined by a GCS≤8 and/or a AIS≥4
Total 134 articles.
2: The reported costs were not specified for the s-TBI cohort
Total 28 articles.
3: The in-hospital costs were non distinguishable from other costs (such as rehabilitation costs)
Total 13 articles.
4: Other reason
Total 4 articles (3 because articles included no original data and 1 because similar data was used that had already been included in another paper from the same author).

**List of excluded articles after full-text screening with reason for exclusion**

Ackland et al. 2016 [1]: 2
Alali et al. 2014 [2]: 3
Andrioti et al. 2013 [3]: 2
Avraham et al. 2017 [4]: 1
Badke et al. 2018 [5]: 2
Baguley et al. 2007 [6]: 1
Baker 2014 [7]: 1
Befeler et al. 2014 [8]: 1
Bennett et al. 1989 [9]: 1
Bhatti et al. 2015 [10]: 2
Bigelow 2009 [11]: 1
Brady et al. 2006 [12]: 1
Brenner et al. 2009 [13]: 1
Brown et al. 2010 [14]: 1
Caballero et al. 2013 [15]: 1
Campbell et al. 2007 [16]: 1
Caro 2011 [17]: 2
Chen et al. 2012 [18]: 1
Christian et al. 2003 [19]: 1
Cifu et al. 1996 [20]: 2
Cifu et al. 2003 [21]: 2
Clement et al. 2013 [22]: 1
Collie et al. 2013 [23]: 3
Collins et al. 2014 [24]: 1
Conner et al. 2010 [25]: 1
Conner et al. 2008 [26]: 1
Corrigan et al. 2010 [27]: 4
Costa et al. 2015 [28]: 1
Cote et al. 2007 [29]: 1
Curtis et al. 2014 [30]: 1
Curtis et al. 2014 [31]: 1
Curtis et al. 2014 [32]: 1
Cuthbert et al. 2011 [33]: 2
Davis et al. 2007 [34]: 1
De Almeida et al. 2016 [35]: 1
Dhakar et al. 2015 [36]: 1
Dikmen et al. 2003 [37]: 2
DiMaggio et al. 2017[38]: 1
Ding et al. 2012 [39]: 2
Dominguez et al. 2001 [40]: 1
Doucet et al. 2011 [41]: 1
Drag et al. 2013 [42]: 1
Du et al. 2014 [43]: 1
Durkin et al. 1998 [44]: 1
Economics 2012 [45]: 4
Effertz et al. 2013 [46]: 1
Ernst et al. 2018 [47]: 1
Faul et al. 2007 [48]: 3
Fineberg et al. 2013 [49]: 1
Fiser et al. 1998 [50]: 1
Flanagan et al. 2005 [51]: 4
Foster et al. 1996 [52]: 1
Fountain et al. 2016 [53]: 1
Frankel et al. 2006 [54]: 2
Friedman et al. 2012 [55]: 1
Fu et al. 2016 [56]: 1
Garcia-Altes et al. 2012 [57]: 1
Garner et al. 2016 [58]: 2
Gates et al. 2017 [59]: 1
Gerrard 2012 [60]: 1
Gilasi et al. 2015 [61]: 1
Graves et al. 2015 [62]: 3
Graves et al. 2013 [63]: 2
Grieve et al. 2016 [64]: 2
Gupta et al. 2016 [65]: 1
Gustavsson et al. 2011 [66]: 1
Han et al. 2015 [67]: 1
Harbrecht et al. 1998 [68]: 1
Harmon et al. 2015 [69]: 1
Harrison et al. 2013 [70]: 2
Ho et al. 2011 [71]: 1
Hoang et al. 2008 [72]: 3
Hode et al. 2017 [73]: 1
Hotz et al. 2000 [74]: 2
Howard et al. 2018 [75]: 2
Hu et al. 2013 [76]: 1
Hyde et al. 2015 [77]: 1
Iapichino et al. 2004 [78]: 1
Ibrahim et al. 2005 [79]: 4
Joseph et al. 2015 [80]: 1
Joseph et al. 2015 [81]: 2
Juhra et al. 2012 [82]: 1
Kalanithi et al. 2011 [83]: 1
Kavosi et al. 2015 [84]: 1
Kayani et al. 2009 [85]: 1
Keenan et al. 2013 [86]: 1
Khan et al. 2002 [87]: 2
King et al. 2009 [88]: 1
Kitchener et al. 2005 [89]: 1
Kreutzer et al. 2001 [90]: 1
Kuczawski et al. 2016 [91]: 1
Kuhn et al. 2016 [92]: 1
Lecky et al. 2016 [93]: 2
Leibson et al. 2012 [94]: 1
Libby et al. 2003 [95]: 1
Lin et al. 2014 [96]: 1
Lundy et al. 2010 [97]: 1
Maercker et al. 2013 [98]: 1
Malmivaara et al. 2011 [99]: 1
Mar et al. 2011 [100]: 1
Marin et al. 2017 [101]: 1
McHugh et al. 2007 [102]: 1
Mitchell et al. 2016 [103]: 1
Mitchell et al. 2013 [104]: 1
Mittelmann et al. 1991 [105]: 1
Mohr et al. 2016 [106]: 1
Moran et al. 2017 [107]: 1
Moreau et al. 2013 [108]: 1
Mujuru et al. 2006 [109]: 1
Murphy et al. 2014 [110]: 1
Nalliah et al. 2014 [111]: 1
Naumann et al. 2015 [112]: 1
Ngwenya et al. 2017 [113]: 3
Nirula et al. 2003 [114]: 3
O’Brien et al. 2013 [115]: 1
Olesen et al. 2012 [116]: 1
Olesen et al. 2008 [117]: 1
Olsen et al. 2016 [118]: 1
Ommaya et al. [119]: 1
Papastrat et al. 1992 [120]: 1
Piatt et al. 2012 [121]: 1
Ponsford et al. 2013 [122]: 3
Race et al. 2004 [123]: 1
Raj et al. 2018 [124]: 3
Ranjan et al. 2006 [125]: 1
Reaven et al. 2009 [126]: 1
Rickels et al. 2010 [127]: 2
Rochette et al. 2008 [128]: 1
Rochette et al. 2009 [129]: 1
Rockhill et al. 2012 [130]: 1
Rosenbaum et al. 2014 [131]: 1
Rudolfson et al. 2018 [132]: 1
Russo et al. 2006 [133]: 1
Salisbury et al. 2017 [134]: 1
Saltzherr et al. 2013 [135]: 3
Sauber-Schatz et al. 2015 [136]: 1
Schneider et al. 2012 [137]: 1
Schneier et al. 2006 [138]: 1
Scholten et al. 2014 [139]: 1
Scholten et al. 2015 [140]: 2
Schulman et al. 2002 [141]: 1
Sears et al. 2013 [142]: 1
Shah et al. 2004 [143]: 2
Shah et al. 2007 [144]: 1
Shi et al. 2014 [145]: 1
Shi et al. 2013 [146]: 1
Shi et al. 2009 [147]: 1
Shih et al. 2013 [148]: 1
Siegel et al. 1995 [149]: 3
Singh et al. 2017 [150]: 1
Slomine et al. 2006 [151]: 2
Smart et al. 2016 [152]: 1
Spain et al. 1998 [153]: 2
Spitz et al. 2016 [154]: 2
Stein et al. 2009 [155]: 3
Stroupe et al. 2013 [156]: 1
Sundbarg et al. 1989 [157]: 1
Sut et al. 2010 [158]: 1
Taylor et al. 2012 [159]: 1
Taylor et al. 2012 [160]: 1
Taylor et al. 2012 [161]: 1
Te et al. 2014 [162]: 1
Thompson et al. 2012 [163]: 1
Tilford en al. 2005 [164]: 1
Tilford et al. 2007 [165]: 1
Tilford et al. 2013 [166]: 1
Tuominen et al. 2012 [167]: 1
van Heugten et al. 2011 [168]: 1
Vangel et al. 2005 [169]: 1
Vespa et al. 2007 [170]: 1
Vitaz et al. 2001 [171]: 3
Wee et al. 2016 [172]: 2
Wei et al. 2005 [173]: 1
Weiss et al. 2010 [174]: 2
Whitlock et al. 1995 [175]: 1
Wrona et al. 2006 [176]: 1
Yang et al. 2011 [177]: 1
Zaloshnja et al. 2012 [178]: 1
Zonfrillo et al. 2016 [179]: 1

**Reference list of exclusions:**

1. Ackland HM, Pilcher DV, Roodenburg OS, McLellan SA, Cameron PA, Cooper DJ. Danger at every rung: Epidemiology and outcomes of ICU-admitted ladder-related trauma. Injury. 2016;47(5):1109-17.

2. Alali AS, Naimark DM, Wilson JR, Fowler RA, Scales DC, Golan E, et al. Economic evaluation of decompressive craniectomy versus barbiturate coma for refractory intracranial hypertension following traumatic brain injury. Critical care medicine. 2014;42(10):2235-43.

3. Andrioti D, Zenonos P, Skitsou A, Kokkori P, Charalambous G. The direct costs of patients with traumatic brain injury in an intensive care unit (ICU) in Cyprus. International Journal of Caring Sciences. 2013;6(2):278-85.

4. Avraham JB, Bhandari M, Frangos SG, Levine DA, Tunik MG, DiMaggio CJ. Epidemiology of paediatric trauma presenting to US emergency departments: 2006-2012. Inj Prev. 2017.

5. Badke GL, Araujo JLV, Miura FK, Guirado VMD, Saade N, Paiva ALC, et al. Analysis of direct costs of decompressive craniectomy in victims of traumatic brain injury. Arquivos De Neuro-Psiquiatria. 2018;76(4):257-64.

6. Baguley IJ, Slewa-Younan S, Heriseanu RE, Nott MT, Mudaliar Y, Nayyar V. The incidence of dysautonomia and its relationship with autonomic arousal following traumatic brain injury. Brain injury. 2007;21(11):1175-81.

7. Baker MS. Casualties of the Global War on Terror and their future impact on health care and society: a looming public health crisis. Mil Med. 2014;179(4):348-55.

8. Befeler AR, Daniels DJ, Helms SA, Klimo P, Jr., Boop F. Head injuries following television-related accidents in the pediatric population. Journal of neurosurgery Pediatrics. 2014;14(4):414-7.

9. Bennett BR, Jacobs LM, Schwartz RJ. Incidence, costs, and DRG-based reimbursement for traumatic brain injured patients: a 3-year experience. J Trauma. 1989;29(5):556-65.

10. Bhatti J, Stevens K, Mir M, Hyder AA, Razzak J. Emergency care of traumatic brain injuries in Pakistan: a multicenter study. BMC Emerg Med. 2015;15 Suppl 2:S12.

11. Bigelow W. HEALTH AND COST OUTCOMES RESULTING FROM TRAUMATIC BRAIN INJURY CAUSED BY NOT WEARING A HELMET, FOR MOTORCYCLE CRASHES-áGÇª. 2009.

12. Brady SL, Darragh M, Escobar NG, O'Neil K, Pape TL-B, Rao N. Persons with disorders of consciousness: Are oral feedings safe/effective? Brain injury. 2006;20(13-14):1329-34.

13. Brenner LA, Carlson NE, Harrison-Felix C, Ashman T, Hammond FM, Hirschberg RE. Self-inflicted traumatic brain injury: Characteristics and outcomes. Brain injury. 2009;23(13-14):991-8.

14. Brown CV, Foulkrod KH, Lopez D, Stokes J, Villareal J, Foarde K, et al. Recombinant factor VIIa for the correction of coagulopathy before emergent craniotomy in blunt trauma patients. The Journal of trauma. 2010;68(2):348-52.

15. Caballero GC, Hughes DW, Maxwell PR, Green K, Gamboa CD, Barthol CA. Retrospective analysis of levetiracetam compared to phenytoin for seizure prophylaxis in adults with traumatic brain injury. Hosp Pharm. 2013;48(9):757-61.

16. Campbell KA, Berger RP, Ettaro L, Roberts MS. Cost-effectiveness of head computed tomography in infants with possible inflicted traumatic brain injury. Pediatrics. 2007;120(2):295-304.

17. Caro D. Towards sustainable traumatic brain injury care systems: healthcare leadership imperatives in Canada. Healthc Manage Forum. 2011;24(1):25-30.

18. Chen A, Bushmeneva K, Zagorski B, Colantonio A, Parsons D, Wodchis WP. Direct cost associated with acquired brain injury in Ontario. BMC neurology. 2012;12:76.

19. Christian WJ, Carroll M, Meyer K, Vitaz TW, Franklin GA. Motorcycle helmets and head injuries in Kentucky, 1995-2000. J Ky Med Assoc. 2003;101:21-6.

20. Cifu DX, Kreutzer JS, Marwitz JH, Rosenthal M, Englander J, High W. Functional outcomes of older adults with traumatic brain injury: a prospective, multicenter analysis. Archives of Physical Medicine and Rehabilitation. 1996;77:883-8.

21. Cifu DX, Kreutzer JS, Kolakowsky-Hayner SA, Marwitz JH, Englander J. The relationship between therapy intensity and rehabilitative outcomes after traumatic brain injury: a multicenter analysis. Archives of physical medicine and rehabilitation. 2003;84(10):1441-8.

22. Clement RC, Carr BG, Kallan MJ, Wolff C, Reilly PM, Malhotra NR. Volume-outcome relationship in neurotrauma care. Journal of neurosurgery. 2013;118(3):687-93.

23. Collie A, Prang KH. Patterns of healthcare service utilisation following severe traumatic brain injury: an idiographic analysis of injury compensation claims data. Injury. 2013;44(11):1514-20.

24. Collins CL, Yeates KO, Pommering TL, Andridge R, Coronado VG, Gilchrist J, et al. Direct medical charges of pediatric traumatic brain injury in multiple clinical settings. Injury Epidemiology. 2014;1(1).

25. Conner KA, Williams LE, McKenzie LB, Shields BJ, Fernandez SA, Smith GA. Pediatric pedestrian injuries and associated hospital resource utilization in the United States, 2003. The Journal of trauma. 2010;68(6):1406-12.

26. Conner KA, Xiang H, Groner JI, Smith GA. Using data linkage to assess the impact of motorized recreational vehicle-related injuries in Ohio. Journal of safety research. 2008;39(5):469-75.

27. Corrigan JD, Selassie AW, Orman JA. The epidemiology of traumatic brain injury. The Journal of head trauma rehabilitation. 2010;25(2):72-80.

28. Costa CK, Dagher JH, Lamoureux J, de GE, Feyz M. Societal cost of traumatic brain injury: A comparison of cost-of-injuries related to biking with and without helmet use. Brain injury. 2015;29(7-8):843-7.

29. Cote MJ, Syam SS, Vogel WB, Cowper DC. A mixed integer programming model to locate traumatic brain injury treatment units in the Department of Veterans Affairs: a case study. Health Care Manag Sci. 2007;10(3):253-67.

30. Curtis K, Chan DL, Lam MK, Mitchell R, King K, Leonard L, et al. The injury profile and acute treatment costs of major trauma in older people in New South Wales. Australas J Ageing. 2014;33(4):264-70.

31. Curtis K, Lam M, Mitchell R, Dickson C, McDonnell K. Major trauma: the unseen financial burden to trauma centres, a descriptive multicentre analysis. Aust Health Rev. 2014;38(1):30-7.

32. Curtis K, Lam M, Mitchell R, Black D, Taylor C, Dickson C, et al. Acute costs and predictors of higher treatment costs of trauma in New South Wales, Australia. Injury. 2014;45(1):279-84.

33. Cuthbert JP, Corrigan JD, Harrison-Felix C, Coronado V, Dijkers MP, Heinemann AW, et al. Factors that predict acute hospitalization discharge disposition for adults with moderate to severe traumatic brain injury. Archives of physical medicine and rehabilitation. 2011;92(5):721-30.

34. Davis KL, Joshi AV, Tortella BJ, Candrilli SD. The direct economic burden of blunt and penetrating trauma in a managed care population. The Journal of trauma. 2007;62(3):622-9.

35. De Almeida CER, De Sousa Filho JL, Dourado JC, Gontijo PAM, Dellaretti MA, Costa BS. Traumatic Brain Injury Epidemiology in Brazil. World neurosurgery. 2016;87:540-7.

36. Dhakar MB, Sivakumar S, Bhattacharya P, Shah A, Basha MM. A retrospective cross-sectional study of the prevalence of generalized convulsive status epilepticus in traumatic brain injury: United States 2002-2010. Seizure. 2015;32:16-22.

37. Dikmen SS, Machamer JE, Powell JM, Temkin NR. Outcome 3 to 5 years after moderate to severe traumatic brain injury. Archives of physical medicine and rehabilitation. 2003;84(10):1449-57.

38. DiMaggio CJ, Avraham JB, Lee DC, Frangos SG, Wall SP. The Epidemiology of Emergency Department Trauma Discharges in the United States. Academic emergency medicine : official journal of the Society for Academic Emergency Medicine. 2017.

39. Ding J, Yuan F, Guo Y, Chen SW, Gao WW, Wang G, et al. A prospective clinical study of routine repeat computed tomography (CT) after traumatic brain injury (TBI). Brain injury. 2012;26:1211-6.

40. Dominguez TE, Chalom R, Costarino J. The severity and cost of child abuse in the pediatric intensive care unit. Journal of Intensive Care Medicine. 2001;16(1):35-41.

41. Doucet JJ, Hill L, Stout P, Bansal V, Lee J, Fortlage D, et al. The unrecognized danger of a new transportation mechanism of injury--pedicabs. Journal of safety research. 2011;42(2):131-5.

42. Drag L, Renninger C, King R, Hoblyn J. Predictors of inpatient and outpatient healthcare utilization in veterans with traumatic brain injury. The Journal of head trauma rehabilitation. 2013;28(1):39-47.

43. Du W, Yang J, Powis B, Zheng X, Ozanne-Smith J, Bilston L, et al. Epidemiological profile of hospitalised injuries among electric bicycle riders admitted to a rural hospital in Suzhou: a cross-sectional study. Inj Prev. 2014;20(2):128-33.

44. Durkin MS, Olsen S, Barlow B, Virella A, Connolly ES, Jr. The epidemiology of urban pediatric neurological trauma: evaluation of, and implications for, injury prevention programs. Neurosurgery. 1998;42(2):300-10.

45. Economics A. The economic cost of spinal cord injury and traumatic brain injury in Australia. Report by Access Economics Pty Limited for The Victorian Neurotrauma. 2012.

46. Effertz T, Mann K. The burden and cost of disorders of the brain in Europe with the inclusion of harmful alcohol use and nicotine addiction. European Neuropsychopharmacology. 2013;23(7):742-8.

47. Ernst G, Qeadan F, Carlson AP. Subcutaneous bone flap storage after emergency craniectomy: cost-effectiveness and rate of resorption. Journal of neurosurgery. 2018:1-7. Epub 2018/01/06. doi: 10.3171/2017.6.jns17943.

48. Faul M, Wald MM, Rutland-Brown W, Sullivent EE, Sattin RW. Using a cost-benefit analysis to estimate outcomes of a clinical treatment guideline: testing theBrain Trauma Foundation guidelines for the treatment of severe traumatic brain injury. The Journal of trauma. 2007;63(6):1271-8.

49. Fineberg NA, Haddad PM, Carpenter L, Gannon B, Sharpe R, Young AH, et al. The size, burden and cost of disorders of the brain in the UK. Journal of Psychopharmacology. 2013;27(9):761-70.

50. Fiser SM, Johnson SB, Fortune JB. Resource utilization in traumatic brain injury: the role of magnetic resonance imaging. Am Surg. 1998;64(11):1088-93.

51. Flanagan SR, Hibbard MR, Gordon WA. The impact of age on traumatic brain injury. Physical medicine and rehabilitation clinics of North America. 2005;16(1):163-77.

52. Foster J. Predicting resource use for patients with traumatic brain injury. AACN Clin Issues. 1996;7(1):168-74.

53. Fountain DM, Kolias AG, Laing RJ, Hutchinson PJ. The financial outcome of traumatic brain injury: a single centre study. British journal of neurosurgery. 2016:1-6.

54. Frankel JE, Marwitz JH, Cifu DX, Kreutzer JS, Englander J, Rosenthal M. A follow-up study of older adults with traumatic brain injury: taking into account decreasing length of stay. Archives of physical medicine and rehabilitation. 2006;87(1):57-62.

55. Friedman J, Reed P, Sharplin P, Kelly P. Primary prevention of pediatric abusive head trauma: A cost audit and cost-utility analysis. Child Abuse & Neglect. 2012;36(11-12):760-70.

56. Fu TS, Jing R, McFaull SR, Cusimano MD. Health & Economic Burden of Traumatic Brain Injury in the Emergency Department. Can J Neurol Sci. 2016;43(2):238-47.

57. Garcia-Altes A, Perez K, Novoa A, Suelves JM, Bernabeu M, Vidal J, et al. Spinal cord injury and traumatic brain injury: a cost-of-illness study. Neuroepidemiology. 2012;39(2):103-8.

58. Garner AA, Mann KP, Fearnside M, Poynter E, Gebski V. The Head Injury Retrieval Trial (HIRT): a single-centre randomised controlled trial of physician prehospital management of severe blunt head injury compared with management by paramedics only. Emergency Medicine Journal. 2016;32:869-75.

59. Gates M, Mallory G, Planchard R, Nothdurft G, Graffeo C, Atkinson J. Triage Patterns of Traumatic Subarachnoid Hemorrhage: Is Referral to a Tertiary Care Center Necessary? World neurosurgery. 2017;100:417-23.

60. Gerrard P. Age-related trends in intracranial injury outcomes. The journal of trauma and acute care surgery. 2012;73(5):1242-6.

61. Gilasi HR, Soori H, Yazdani S, Taheri TP. Fall-Related Injuries in Community-Dwelling Older Adults in Qom Province, Iran, 2010-2012. Archives of trauma research. 2015;4(1):e22925.

62. Graves JM, Rivara FP, Vavilala MS. Health Care Costs 1 Year After Pediatric Traumatic Brain Injury. American journal of public health. 2015;105(10):e35-e41.

63. Graves JM, Sears JM, Vavilala MS, Rivara FP. The burden of traumatic brain injury among adolescent and young adult workers in Washington State. Journal of safety research. 2013;45:133-9.

64. Grieve R, Sadique Z, Gomes M, Smith M, Lecky FE, Hutchinson PJ, et al. An evaluation of the clinical and cost-effectiveness of alternative care locations for critically ill adult patients with acute traumatic brain injury. British journal of neurosurgery. 2016;30(4):388-96.

65. Gupta DK, Bisht A, Batra P, Mathur P, Mahapatra AK. A cost effectiveness based safety and efficacy study of resterilized intra-parenchymal catheter based intracranial pressure monitoring in developing world. Asian journal of neurosurgery. 2016;11(4):416-20.

66. Gustavsson A, Svensson M, Jacobi F, Allgulander C, Alonso J, Beghi E, et al. Cost of disorders of the brain in Europe 2010. European neuropsychopharmacology : the journal of the European College of Neuropsychopharmacology. 2011;21(10):718-79.

67. Han GM, Newmyer A, Qu M. Seat belt use to save face: impact on drivers' body region and nature of injury in motor vehicle crashes. Traffic Inj Prev. 2015;16(6):605-10.

68. Harbrecht BG, Moraca RJ, Saul M, Courcoulas AP. Percutaneous endoscopic gastrostomy reduces total hospital costs in head-injured patients. Am J Surg. 1998;176(4):311-4.

69. Harmon KJ, Marshall SW, Proescholdbell SK, Naumann RB, Waller AE. Motorcycle crash-related emergency department visits and hospitalizations for traumatic brain injury in North Carolina. The Journal of head trauma rehabilitation. 2015;30(3):175-84.

70. Harrison DA, Prabhu G, Grieve R, Harvey SE, Sadique MZ, Gomes M, et al. Risk Adjustment In Neurocritical care (RAIN)--prospective validation of risk prediction models for adult patients with acute traumatic brain injury to use to evaluate the optimum location and comparative costs of neurocritical care: a cohort study. Health technology assessment (Winchester, England). 2013;17(23):vii-350.

71. Ho KM, Honeybul S, Lind CR, Gillett GR, Litton E. Cost-effectiveness of decompressive craniectomy as a lifesaving rescue procedure for patients with severe traumatic brain injury. The Journal of trauma. 2011;71(6):1637-44.

72. Hoang HT, Pham TL, Vo TT, Nguyen PK, Doran CM, Hill PS. The costs of traumatic brain injury due to motorcycle accidents in Hanoi, Vietnam. Cost Eff Resour Alloc. 2008;6:17.

73. Hode L, Madougou S, Fatigba HO, Hounnou P, Ebassa K, Hans Moevi AA, et al. The Direct Cost of Treatment of Traumatic Brain Injury in a Sub-Saharan African Country (Benin). World neurosurgery. 2017;99:210-3.

74. Hotz GA, Stewart KJ, Petrin D, Villanueva PA, Cohn SM, Nedd KJ, et al. Neurobehavioural outcomes of penetrating and tangential gunshot wounds to the head. Brain injury. 2000;14(7):649-57.

75. Howard SW, Zhang Z, Buchanan P, Bernell SL, Williams C, Pearson L, et al. The cost of a pediatric neurocritical care program for traumatic brain injury: a retrospective cohort study. BMC Health Services Research. 18(1):20.

76. Hu J, Ugiliweneza B, Meyer K, Lad SP, Boakye M. Trend and geographic analysis for traumatic brain injury mortality and cost based on MarketScan database. Journal of neurotrauma. 2013;30(20):1755-61.

77. Hyde GA, Savage SA, Zarzaur BL, Hart-Hyde JE, Schaefer CB, Croce MA, et al. Early tracheostomy in trauma patients saves time and money. Injury. 2015;46(1):110-4.

78. Iapichino G, Radrizzani D, Simini B, Rossi C, Albicini M, Ferla L, et al. Effectiveness and efficiency of intensive care medicine: variable costs in different diagnosis groups. Acta Anaesthesiol Scand. 2004;48(7):820-6.

79. Ibrahim M, Abdullah M, Naing L, ... Cost effectiveness analysis of using multiple monitoring modalities in treating severe traumatic brain injury (CESTBI). Journal of-áGÇª. 2005.

80. Joseph B, Pandit V, Haider AA, Kulvatunyou N, Zangbar B, Tang A, et al. Improving Hospital Quality and Costs in Nonoperative Traumatic Brain Injury: The Role of Acute Care Surgeons. JAMA surgery. 2015;150(9):866-72.

81. Joseph B, Haider AA, Pandit V, Tang A, Kulvatunyou N, O'Keeffe T, et al. Changing paradigms in the management of 2184 patients with traumatic brain injury. Annals of surgery. 2015;262(3):440-8.

82. Juhra C, Wiesk+¦tter B, Chu K, Trost L, Weiss U, Messerschmidt M, et al. Bicycle accidents GÇô Do we only see the tip of the iceberg?: A prospective multi-centre study in a large German city combining medical and police data. Injury. 2012;43(12):2026-34

83. Kalanithi P, Schubert RD, Lad SP, Harris OA, Boakye M. Hospital costs, incidence, and inhospital mortality rates of traumatic subdural hematoma in the United States: Clinical article. Journal of neurosurgery. 2011;115(5):1013-8.

84. Kavosi Z, Jafari A, Hatam N, Enaami M. The economic burden of traumatic brain injury due to fatal traffic accidents in shahid rajaei trauma hospital, shiraz, iran. Archives of trauma research. 2015;4(1):e22594.

85. Kayani NA, Homan S, Yun S, Zhu BP. Health and economic burden of traumatic brain injury: Missouri, 2001-2005. Public Health Rep. 2009;124(4):551-60.

86. Keenan HT, Murphy NA, Staheli R, Savitz LA. Healthcare utilization in the first year after pediatric traumatic brain injury in an insured population. Journal of Head Trauma Rehabilitation. 2013;28(6):426-32.

87. Khan S, Khan A, Feyz M. Decreased Length of stay, cost savings and descriptive findings of enhanced patient care resulting from and integrated traumatic brain injury programme. Brain injury. 2002;16(6):537-54.

88. King DA, Hume PA, Milburn P, Gianotti S. Rugby league injuries in New Zealand: A review of 8 years of Accident Compensation Corporation injury entitlement claims and costs. British journal of sports medicine. 2009;43(8):595-602.

89. Kitchener M, Ng T, Grossman B, Harrington C. Medicaid waiver programs for traumatic brain and spinal cord injury. J Health Soc Policy. 2005;20(3):51-66.

90. Kreutzer JS, Kolakowsky-Hayner SA, Ripley D, Cifu DX, Rosenthal M, Bushnik T, et al. Charges and lengths of stay for acute and inpatient rehabilitation treatment of traumatic brain injury 1990-1996. Brain injury. 2001;15(9):763-74.

91. Kuczawski M, Stevenson M, Goodacre S, Teare MD, Ramlakhan S, Morris F, et al. Should all anticoagulated patients with head injury receive a CT scan? Decision-analysis modelling of an observational cohort. BMJ Open. 2016;6(12):e013742.

92. Kuhn EN, Warmus BA, Davis MC, Oster RA, Guthrie BL. Identification and Cost of Potentially Avoidable Transfers to a Tertiary Care Neurosurgery Service: A Pilot Study. Neurosurgery. 2016;79(4):541-8.

93. Lecky F, Russell W, Fuller G, McClelland G, Pennington E, Goodacre S, et al. The head injury transportation straight to neurosurgery (HITS-NS) randomised trial: A feasibility study. Health Technology Assessment. 2016;20:1-xxvii.

94. Leibson CL, Brown AW, Hall LK, Ransom JE, Mandrekar J, Osler TM, et al. Medical care costs associated with traumatic brain injury over the full spectrum of disease: a controlled population-based study. Journal of neurotrauma. 2012;29(11):2038-49.

95. Libby AM, Sills MR, Thurston NK, Orton HD. Costs of childhood physical abuse: comparing inflicted and unintentional traumatic brain injuries. Pediatrics. 2003;112(1 Pt 1):58-65.

96. Lin CM, Li CY. Assessment of medical resource utilization for Taiwanese children hospitalized for intracranial injuries. Injury. 2014;45(4):690-5.

97. Lundy CT, Woodthorpe C, Hedderly TJ, Chandler C, Lasoye T, McCormick D. Outcome and cost of childhood brain injury following assault by young people. Emergency Medicine Journal. 2010;27(9):659-62.

98. Maercker A, Perkonigg A, Preisig M, Schaller K, Weller M. The costs of disorders of the brain in Switzerland: An update from the European Brain Council Study for 2010. Swiss Medical Weekly. 2013;143(no pagination).

99. Malmivaara K, Kivisaari R, Hernesniemi J, Siironen J. Cost-effectiveness of decompressive craniectomy in traumatic brain injuries. European journal of neurology. 2011;18(4):656-62.

100. Mar J, Arrospide A, Begiristain JM, Larranaga I, Elosegui E, Oliva-Moreno J. The impact of acquired brain damage in terms of epidemiology, economics and loss in quality of life. BMC neurology. 2011;11:46

101. Marin JR, Weaver MD, Mannix RC. Burden of USA hospital charges for traumatic brain injury. Brain injury. 2017;31(1):24-31.

102. McHugh JC, Sobocki P, Murphy RP. Cost of disorders of the Brain in Ireland. Irish Medical Journal. 2007;100(7).

103. Mitchell RJ, Bambach MR. Personal injury recovery cost of pedestrian-vehicle collisions in New South Wales, Australia. Traffic Inj Prev. 2016;17(5):508-14.

104. Mitchell RJ, Curtis K, Holland AJ, Balogh ZJ, Evans J, Wilson KL. Acute costs and predictors of higher treatment costs for major paediatric trauma in New South Wales, Australia. Journal of Paediatrics and Child Health. 2013;49(7):557-63.

105. Mittelmann M, Urso J, Baldwin B, Finnerty DC. Workers Compensation cases with traumatic brain injury: an insurance carrier's analysis of care, costs, and outcomes. J Insur Med. 1991;23(1):55-63.

106. Mohr NM, Harland KK, Shane DM, Miller SL, Torner JC, Newgard CD. Potentially Avoidable Pediatric Interfacility Transfer Is a Costly Burden for Rural Families: A Cohort Study. Academic Emergency Medicine. 2016;23(8):885-94.

107. Moran D, Shrime MG, Nang S, Vycheth I, Vuthy D, Hong R, et al. Cost-Effectiveness of Craniotomy for Epidural Hematomas at a Major Government Hospital in Cambodia. World journal of surgery. 2017.

108. Moreau JF, Fink EL, Hartman ME, Angus DC, Bell MJ, Linde-Zwirble WT, et al. Hospitalizations of children with neurologic disorders in the United States. Pediatric critical care medicine : a journal of the Society of Critical Care Medicine and the World Federation of Pediatric Intensive and Critical Care Societies. 2013;14(8):801-10. 109. Mujuru P, Singla L, Helmkamp J, Bell J, Hu W. Evaluation of the burden of logging injuries using West Virginia workers' compensation claims data from 1996 to 2001. Am J Ind Med. 2006;49(12):1039-45.

110. Murphy TE, Baker DI, Leo-Summers LS, Tinetti ME. Trends in Fall-Related Traumatic Brain Injury among Older Persons in Connecticut from 2000-2007. J Gerontol Geriatr Res. 2014;3(4).

111. Nalliah RP, Anderson IM, Lee MK, Rampa S, Allareddy V, Allareddy V. Epidemiology of hospital-based emergency department visits due to sports injuries. Pediatr Emerg Care. 2014;30(8):511-5.

112. Naumann RB, Marshall SW, Proescholdbell SK, Austin A, Creppage K. Impact of North Carolina's motorcycle helmet law on hospital admissions and charges for care of traumatic brain injuries. North Carolina medical journal. 2015;76(2):70-5.

113. Ngwenya LB, Suen CG, Tarapore PE, Manley GT, Huang MC. Safety and cost efficiency of a restrictive transfusion protocol in patients with traumatic brain injury. Journal of neurosurgery. 2017:1-8.

114. Nirula R, Kaufman R, Tencer A. Traumatic brain injury and automotive design: making motor vehicles safer. The Journal of trauma. 2003;55(5):844-8.

115. O'Brien JE, Dumas HM. Hospital length of stay, discharge disposition, and reimbursement by clinical program group in pediatric post-acute rehabilitation. Journal of Pediatric Rehabilitation Medicine. 2013;6(1):29-34.

116. Olesen J, Gustavsson A, Svensson M, Wittchen HU, Jonsson B. The economic cost of brain disorders in Europe. European journal of neurology. 2012;19(1):155-62.

117. Olesen J, Sobscki P, Truelsen T, Sestoft D, Jonsson B. Cost of disorders of the brain in Denmark. Nordic Journal of Psychiatry. 2008;62(2):114-20.

118. Olsen CS, Thomas AM, Singleton M, Gaichas AM, Smith TJ, Smith GA, et al. Motorcycle helmet effectiveness in reducing head, face and brain injuries by state and helmet law. Inj Epidemiol. 2016;3(1):8.

119. Ommaya AK, Ommaya AK, Dannenberg AL, Salazar AM. Causation, incidence, and costs of traumatic brain injury in the U.S. military medical system. J Trauma. 1996;40(2):211-7.

120. Papastrat LA. Outcome and value following brain injury: A financial provider's perspective. Journal of Head Trauma Rehabilitation. 1992;7(4):11-23.

121. Piatt JH, Jr., Neff DA. Hospital care of childhood traumatic brain injury in the United States, 1997-2009: a neurosurgical perspective. Journal of neurosurgery Pediatrics. 2012;10(4):257-67.

122. Ponsford JL, Spitz G, Cromarty F, Gifford D, Attwood D. Costs of care after traumatic brain injury. Journal of neurotrauma. 2013;30(17):1498-505.

123. Race MC, Carlile MC. Motorcycle-related injuries: the high costs of riding. Tex Med. 2004;100(10):56-63.

124. Raj R, Bendel S, Reinikainen M, Hoppu S, Luoto T, Ala-Kokko T, et al. Temporal Trends in Healthcare Costs and Outcome Following ICU Admission After Traumatic Brain Injury. Critical care medicine. 46(4):e302-e9.

125. Ranjan D, Schmonsky K, Johnston T, Jeon H, Bouneva I, Erway E. Financial analysis of potential donor management at a Medicare-approved transplant hospital. American Journal of Transplantation. 2006;6(1):199-204.

126. Reaven NL, Lovett JE, Funk SE. Brain injury and fever: Hospital length of stay and cost outcomes. Journal of Intensive Care Medicine. 2009;24(2):131-9.

127. Rickels E, von WK, Wenzlaff P. Head injury in Germany: A population-based prospective study on epidemiology, causes, treatment and outcome of all degrees of head-injury severity in two distinct areas. Brain injury. 2010;24(12):1491-504..

128. Rochette LM, Conner KA, Smith GA. The Medical and Economic Impact of Motorized Recreational Vehicle-Related Traumatic Brain Injury in Ohio. 2008.

129. Rochette LM, Conner KA, Smith GA. The contribution of traumatic brain injury to the medical and economic outcomes of motor vehicle-related injuries in Ohio. Journal of safety research. 2009;40(5):353-8.

130. Rockhill CM, Jaffe K, Zhou C, Fan MY, Katon W, Fann JR. Health care costs associated with traumatic brain injury and psychiatric illness in adults. Journal of neurotrauma. 2012;29(6):1038-46. 131. Rosenbaum BP, Kelly ML, Kshettry VR, Weil RJ. Neurologic disorders, in-hospital deaths, and years of potential life lost in the USA, 1988-2011. J Clin Neurosci. 2014;21(11):1874-80.

132. Rudolfson N, Dewan MC, Park KB, Shrime MG, Meara JG, Alkire BC. The economic consequences of neurosurgical disease in low- and middle-income countries. Journal of neurosurgery. 2018:1-8. Epub 2018/05/19. doi: 10.3171/2017.12.jns17281.
133. Russo CA, Steiner C. Hospital Admissions for Traumatic Brain Injuries, 2004: Statistical Brief #27. 2006. doi: NBK63511 [bookaccession].

134. Salisbury DB, Driver SJ, Reynolds M, Bennett M, Petrey LB, Warren AM. Hospital-Based Health Care After Traumatic Brain Injury. Archives of physical medicine and rehabilitation. 2017;98(3):425-33.

135. Saltzherr TP, Goslings JC, Bakker FC, Beenen LFM, Olff M, Meijssen K, et al. Cost-effectiveness of trauma CT in the trauma room versus the radiology department: The REACT trial. European Radiology. 2013;23(1):148-55.

136. Sauber-Schatz EK, Thomas AM, Cook LJ. Motor Vehicle Crashes, Medical Outcomes, and Hospital Charges Among Children Aged 1-12 Years - Crash Outcome Data Evaluation System, 11 States, 2005-2008. Morbidity and mortality weekly report Surveillance summaries (Washington, DC : 2002). 2015;64(8):1-32.

137. Schneider EB, Hirani SA, Hambridge HL, Haut ER, Carlini AR, Castillo RC, et al. Beating the weekend trend: increased mortality in older adult traumatic brain injury (TBI) patients admitted on weekends. The Journal of surgical research. 2012;177(2):295-300.

138. Schneier AJ, Shields BJ, Hostetler SG, Xiang H, Smith GA. Incidence of pediatric traumatic brain injury and associated hospital resource utilization in the United States. Pediatrics. 2006;118(2):483-92.

139. Scholten AC, Haagsma JA, Panneman MJ, van Beeck EF, Polinder S. Traumatic brain injury in the Netherlands: incidence, costs and disability-adjusted life years. PloS one. 2014;9(10):e110905. doi: 10.1371/journal.pone.0110905
140. Scholten AC, Polinder S, Panneman MJ, van Beeck EF, Haagsma JA. Incidence and costs of bicycle-related traumatic brain injuries in the Netherlands. Accid Anal Prev. 2015;81:51-60.

141. Schulman J, Sacks J, Provenzano G. State level estimates of the incidence and economic burden of head injuries stemming from non-universal use of bicycle helmets. Injury Prevention. 2002;8(1):47-52.

142. Sears JM, Blanar L, Bowman SM, Adams D, Silverstein BA. Predicting work-related disability and medical cost outcomes: estimating injury severity scores from workers' compensation data. J Occup Rehabil. 2013;23(1):19-31.

143. Shah MK, Al-Adawi S, Dorvlo AS, Burke DT. Functional outcomes following anoxic brain injury: a comparison with traumatic brain injury. Brain injury. 2004;18(2):111-7.

144. Shah MK, Carayannopoulos AG, Burke DT, Al-Adawi S. A comparison of functional outcomes in hypoxia and traumatic brain injury: a pilot study. J Neurol Sci. 2007;260(1-2):95-9.

145. Shi HY, Hwang SL, Lee IC, Chen IT, Lee KT, Lin CL. Trends and outcome predictors after traumatic brain injury surgery: a nationwide population-based study in Taiwan. Journal of neurosurgery. 2014;121(6):1323-30.

146. Shi H-Y, Hwang S-L, Lee K-T, Lin C-L. Temporal trends and volume-outcome associations after traumatic brain injury: A 12-year study in Taiwan: Clinical article. Journal of neurosurgery. 2013;118(4):732-8.

147. Shi J, Xiang H, Wheeler K, Smith GA, Stallones L, Groner J, et al. Costs, mortality likelihood and outcomes of hospitalized US children with traumatic brain injuries. Brain injury. 2009;23(7):602-11.

148. Shih CC, Lee HH, Chen TL, Tsai CC, Lane HL, Chiu WT, et al. Reduced use of emergency care and hospitalization in patients with traumatic brain injury receiving acupuncture treatment (Provisional abstract). Evidence Based Complementary and Alternative Medicine. 2013:262039.

149. Siegel JH. The effect of associated injuries, blood loss, and oxygen debt on death and disability in blunt traumatic brain injury: the need for early physiologic predictors of severity. J Neurotrauma. 1995;12(4):579-90.

150. Singh R, Sinha S, Bill A, Turner-Stokes L. Unmet need for specialised rehabilitation following neurosurgery: can we maximise the potential cost-benefits? British journal of neurosurgery. 2017;31(2):249-53.

151. Slomine BS, McCarthy ML, Ding R, MacKenzie EJ, Jaffe KM, Aitken ME, et al. Health care utilization and needs after pediatric traumatic brain injury. Pediatrics. 2006;117(4):e663-e74.

152. Smart BJ, Haring RS, Asemota AO, Scott JW, Canner JK, Nejim BJ, et al. Tackling causes and costs of ED presentation for American football injuries: a population-level study. Am J Emerg Med. 2016;34(7):1198-204.

153. Spain DA, McIlvoy LH, Fix SE, Carillo EH, Boaz PW, Harpring JE, et al. Effect of a clinical pathway for severe traumatic brain injury on resource utilization. Journal of Trauma - Injury, Infection and Critical Care. 1998;45(1):101-5.

154. Spitz G, McKenzie D, Attwood D, Ponsford JL. Cost prediction following traumatic brain injury: model development and validation. J Neurol Neurosurg Psychiatry. 2016;87(2):173-80. d

155. Stein DM, Dutton RP, Kramer ME, Scalea TM. Reversal of coagulopathy in critically ill patients with traumatic brain injury: recombinant factor VIIa is more cost-effective than plasma. The Journal of trauma. 2009;66(1):63-72.

156. Stroupe KT, Smith BM, Hogan TP, St Andre JR, Pape T, Steiner ML, et al. Healthcare utilization and costs of Veterans screened and assessed for traumatic brain injury. Journal of rehabilitation research and development. 2013;50(8):1047-68.

157. Sundbarg G, Norlund A, Nordstrom CH, Messeter K. Severe traumatic brain lesions in Sweden. Part 3: Economic aspects of aggressive neurosurgical intensive care. Brain injury. 1989;3(3):283-93.

158. Sut N, Memis D. Intensive care cost and survival analyses of traumatic brain injury. Ulus Travma Acil Cerrahi Derg. 2010;16(2):149-54.

159. Taylor BC, Hagel EM, Carlson KF, Cifu DX, Cutting A, ... A.(2012). Prevalence and costs of co-occurring traumatic brain injury with and without psychiatric disturbance and pain among Afghanistan and Iraq war-áGÇª. Medical care.

160. Taylor C, Jan S, Curtis K, Tzannes A, Li Q, Palmer C, et al. The cost-effectiveness of physician staffed Helicopter Emergency Medical Service (HEMS) transport to a major trauma centre in NSW, Australia. Injury. 2012;43(11):1843-9.

161. Taylor BC, Hagel EM, Carlson KF, Cifu DX, Cutting A, Bidelspach DE, et al. Prevalence and costs of co-occurring traumatic brain injury with and without psychiatric disturbance and pain among Afghanistan and Iraq War Veteran V.A. users. Medical care. 2012;50(4):342-6.

162. Te AB, Brown P, Tobias M, Ameratunga S, Barker-Collo S, Theadom A, et al. Cost of traumatic brain injury in New Zealand: evidence from a population-based study. Neurology. 2014;83(18):1645-52.

163. Thompson HJ, Weir S, Rivara FP, Wang J, Sullivan SD, Salkever D, et al. Utilization and costs of health care after geriatric traumatic brain injury. Journal of neurotrauma. 2012;29(10):1864-71.

164. Tilford JM, Aitken ME, Anand KJS, Green JW, Goodman AC, Parker JG, et al. Hospitalizations for critically ill children with traumatic brain injuries: A longitudinal analysis. Critical care medicine. 2005;33(9):2074-81.

165. Tilford JM, Aitken ME, Goodman AC, Adelson PD. Measuring the cost-effectiveness of technologic change in the treatment of pediatric traumatic brain injury. The Journal of trauma. 2007;63(6 Suppl):S113-S20.

166. Tilford JM, Porter A, Boyd M, Pullman M. Hospitalizations and Medical Care Costs of Serious Traumatic Brain Injuries, Spinal Cord Injuries and Traumatic Amputations.

167. Tuominen R, Joelsson P, Tenovuo O. Treatment costs and productivity losses caused by traumatic brain injuries. Brain injury. 2012;26(13-14):1697-701.

168. van Heugten CM, Geurtsen GJ, Derksen RE, Martina JD, Geurts AC, Evers SM. Intervention and societal costs of residential community reintegration for patients with acquired brain injury: a cost-analysis of the Brain Integration Programme. Journal of rehabilitation medicine. 2011;43(7):647-52.

169. Vangel SJ, Jr., Rapport LJ, Hanks RA, Black KL. Long-term medical care utilization and costs among traumatic brain injury survivors. American journal of physical medicine & rehabilitation. 2005;84(3):153-60.

170. Vespa PM, Miller C, Hu X, Nenov V, Buxey F, Martin NA. Intensive care unit robotic telepresence facilitates rapid physician response to unstable patients and decreased cost in neurointensive care. Surg Neurol. 2007;67(4):331-7.

171. Vitaz TW, Mcilvoy L, Raque GH, Spain D, Shields CB. Development and implementation of a clinical pathway for severe traumatic brain injury. Journal of Trauma - Injury, Infection and Critical Care. 2001;51(2):369-75.

172. Wee JZ, Yang YR, Lee QY, Cao K, Chong CT. Demographic profile and extent of healthcare resource utilisation of patients with severe traumatic brain injury: still a major public health problem. Singapore medical journal. 2016;57(9):491-6.

173. Wei W, Sambamoorthi U, Crystal S, Findley PA. Mental illness, traumatic brain injury, and medicaid expenditures. Archives of physical medicine and rehabilitation. 2005;86(5):905-11.

174. Weiss H, Agimi Y, Steiner C. Youth motorcycle-related hospitalizations and traumatic brain injuries in the United States in 2006. Pediatrics. 2010;126(6):1141-8.

175. Whitlock JA, Jr., Hamilton BB. Functional outcome after rehabilitation for severe traumatic brain injury. Arch Phys Med Rehabil. 1995;76(12):1103-12.

176. Wrona RM. The use of state workers' compensation administrative data to identify injury scenarios and quantify costs of work-related traumatic brain injuries. Journal of safety research. 2006;37(1):75-81.

177. Yang CC, Shih NC, Chang WC, Huang SK, Chien CW. Long-term medical utilization following ventilator-associated pneumonia in acute stroke and traumatic brain injury patients: a case-control study. BMC Health Serv Res. 2011;11:289.

178. Zaloshnja E, Miller TR, Coben J, Steiner C. How often do catastrophic injury victims become medicaid recipients? Medical care. 2012;50(6):513-9.

179. Zonfrillo MR, Zaniletti I, Hall M, Fieldston ES, Colvin JD, Bettenhausen JL, et al. Socioeconomic Status and Hospitalization Costs for Children with Brain and Spinal Cord Injury. The Journal of pediatrics. 2016;169:250-5.
