# Supplementary material for: In-hospital costs after severe traumatic brain injury: A systematic review and quality assessment
Source: PLoS One. 2019 May 9;14(5):e0216743. doi: 10.1371/journal.pone.0216743 (PMC6508680; doi:10.1371/journal.pone.0216743)
Supplement: S2 Appendix — (DOCX) [file pone.0216743.s002.docx]

**S2 Appendix. Quality Assessment Information**

| **Quality Assessment Checklist** | | | | Reference # | 1 | 2 | 3 | Score (%) |
| --- | --- | --- | --- | --- | --- | --- | --- | --- |
| Item | # | Study details | | |  |  |  |  |
| Study details | 1 | Is a description of study objectives and/or study questions provided? | | |  |  |  |  |
|  | 2 | Are all relevant aspects of the study setting provided? | | |  |  |  |  |
|  | 3 | Is there a statement about the broader context or relevance for health policy or practice decisions? | | |  |  |  |  |
|  | Subtotal score (N/3) | | | |  |  |  |  |
| Population | 4 | Is there a description of base population and/or subgroups? | | |  |  |  |  |
|  | 5 | * Was a clear definition of illness provided? | | |  |  |  |  |
|  | 6 | Is there a description of TBI severity? | | |  |  |  |  |
|  | Subtotal score (N/4) | | | |  |  |  |  |
| Clinical data | 7 | * Were hospital activity data sources carefully described? | | |  |  |  |  |
|  | 8 | Were hospital activity data appropriately assessed and mentioned? | | |  |  |  |  |
|  | 9 | Was outcome data presented in the study? | | |  |  |  |  |
|  | Subtotal score (N/4) | | | |  |  |  |  |
| Cost data | 10 | | * Were cost data sources carefully described? | |  |  |  |  |
|  | 11 | | * Are the reference year and currency provided? | |  |  |  |  |
|  | 12 | | * Were unit costs appropriately valued? | |  |  |  |  |
|  | 13 | | * Were hospital costs sufficiently disaggregated? | |  |  |  |  |
|  | 14 | | * Were the design and methods of costs analysis carefully described | |  |  |  |  |
|  | 15 | | Were the major assumptions tested in a sensibility analysis | |  |  |  |  |
|  | Subtotal score (N/11) | | | |  |  |  |  |
| Methodology | 16 | | Were study limitations carefully described using study objective, methods and results? | |  |  |  |  |
|  | 17 | | Was there a comparison of the findings with current knowledge? | |  |  |  |  |
|  | 18 | | Was the presentation of study results consistent with the methodology of the study? | |  |  |  |  |
|  | 19 | | Is the source of funding and are possible conflicts of interest mentioned? | |  |  |  |  |
|  | Subtotal score (N/4) | | | |  |  |  |  |
| Total score (%) | | | | |  |  |  |  |

Items designated with a * count double.

Note: For this study two important items for costs research were intentionally left out of the final checklist: (1) the study cost perspective because we only focussed on in-hospital costs, (2) discounting costs since these are considered not relevant for short term in-hospital costs.

**Quality Assessment Checklist Scoring Manual**

| ***Study details*** | ***How to score*** |
| --- | --- |
| 1. Is a description of study objectives and/or study questions provided? | If yes and satisfied: score 1 If yes but not satisfied: score 0.5 If no: score 0 |
| 2. Are all relevant aspects of the study setting provided? | If yes: score 1 If described but considered not clear: score 0.5 If not provided: score 0 |
| 3.Is there a statement about the broader context or relevance for health policy or practice decisions? | If yes and satisfied: score 1 If yes but not satisfied: score 0.5 If no: score 0 |
| ***Population*** | |
| 4. Is there a description of base population and/or subgroups? | If yes and satisfied: score 1 If yes but not satisfied: score 0.5 If no: score 0 |
| 5. * Was a clear definition of the illness provided? | If yes and specified: score 2 If yes, but only mentioned: score 1 If no: score 0 |
| 6. Is there a description of TBI severity? | If yes and specified: score 1 If yes, but only mentioned: score 0.5 If no: score 0 |
| ***Clinical data*** | |
| 7. * Were hospital activity data sources carefully described? | If yes and specified: score 2 If yes, but only mentioned: score 1 If no: score 0 |
| 8. Were hospital activity data appropriately assessed and mentioned? | If yes and satisfied: score 1 If yes but not satisfied: score 0.5 If no: score 0 |
| 9. Was outcome data presented in the study? | If yes: score 1 If yes but only very few: score 0.5 If no: score 0 |
| ***Cost data*** | |
| 10. * Were cost data sources carefully described? | If yes and specified: score 2 If yes, but only mentioned: score 1 If no: score 0 |
| 11. * Are the reference year and currency provided? | If yes: score 2 If only currency/reference year: score 1 If no: score 0 |
| 12. * Were unit costs appropriately valued? | If yes and satisfied: score 2 If yes but not satisfied: score 1 If no: score 0 |
| 13. * Were hospital costs sufficiently disaggregated? | If yes and satisfied: score 2 If yes but not satisfied: score 1 If no: score 0 |
| 14. * Were the design and methods of costs analysis carefully described? | If yes and satisfied: score 2 If yes but not satisfied: score 1 If no: score 0 |
| 15. Were the major assumptions tested in a sensitivity analysis? | If yes: score 1  If no: score 0 |
| ***Methodology*** | |
| 16. Were study limitations carefully described using study objective, methods and results? | If yes and satisfied: score 1 If yes but not satisfied: score 0.5 If no: score 0 |
| 17. Was there a comparison of the findings with current knowledge? | If yes and satisfied: score 1 If yes but not satisfied: score 0.5 If no: score 0 |
| 18. Was the presentation of study results consistent with the methodology of the study? | If yes and satisfied: score 1 If yes but not satisfied: score 0.5 If no: score 0 |
| 19. Is the source of funding and are possible conflicts of interest mentioned? | If yes: score 1 If funding or possible conflicts mentioned: score 0.5 If no: score 0 |

**Making of the Quality Assessment Checklist**

| ***Study details*** | ***Source*** |
| --- | --- |
| 1. Is a description of study objectives and/or study questions provided? | CHEERS ^1, 2^ |
| 2. Are all relevant aspects of the study setting provided? | CHEERS ^1, 2^ |
| 3.Is there a statement about the broader context or relevance for health policy or practice decisions? | CHEERS ^1, 2^ |
| ***Population*** | |
| 4. Is there a description of base population and/or subgroups? | CHEERS ^1, 2^ |
| 5. * Was a clear definition of the illness provided? | Costa ^3^ and Molinier ^4^ |
| 6. Is there a description of TBI severity? | - |
| ***Clinical data*** | |
| 7. * Were hospital activity data sources carefully described? | Costa ^3^ and Molinier ^4^  Also mentioned in CHEERS ^1, 2^ |
| 8. Were hospital activity data appropriately assessed and mentioned? | Costa ^3^ and Molinier ^4^  Also mentioned in CHEERS ^1, 2^ |
| 9. Was outcome data presented in the study? | CHEERS ^1, 2^ |
| ***Cost data*** | |
| 10. * Were cost data sources carefully described? | Polinder et al. ^5^ Also mentioned in CHEERS ^1, 2^ |
| 11. * Are the reference year and currency provided? | CHEERS ^1, 2^ |
| 12. * Were unit costs appropriately valued? | Costa ^3^ and Molinier ^4^  Also mentioned in CHEERS ^1, 2^ |
| 13. * Were hospital costs sufficiently disaggregated? | Costa ^3^  Also mentioned in CHEERS ^1, 2^ |
| 14. * Were the design and methods of costs analysis carefully described? | CHEERS ^1, 2^ |
| 15. Were the major assumptions tested in a sensitivity analysis? | Costa ^3^ and Molinier ^4^  Also mentioned in CHEERS ^1, 2^ |
| ***Methodology*** | |
| 16. Were study limitations carefully described using study objective, methods and results? | Polinder et al. ^5^ Also mentioned in CHEERS ^1, 2^ |
| 17. Was there a comparison of the findings with current knowledge? | Polinder et al. ^5^ Also mentioned in CHEERS ^1, 2^ |
| 18. Was the presentation of study results consistent with the methodology of the study? | Costa ^3^ and Molinier ^4^ |
| 19. Is the source of funding and are possible conflicts of interest mentioned? | CHEERS ^1, 2^ |

CHEERS = Consolidated Health Economic Evaluation Reporting Standards (CHEERS) statement.
Note: Checklists from Costa ^3^ and Molinier ^4^ are based on Drummond ^6^.

**References:**

1. Husereau D, Drummond M, Petrou S, Carswell C, Moher D, Greenberg D, et al. Consolidated health economic evaluation reporting standards (CHEERS) statement. BMJ. 2013;346:f1049
2. Husereau D., Drummond M., Petrou S., Carswell C., Moher D., Greenberg D. et al. Consolidated Health Economic Evaluation Reporting Standards (CHEERS)--explanation and elaboration: a report of the ISPOR Health Economic Evaluation Publication Guidelines Good Reporting Practices Task Force. Value Health. 2005. 16, 231-250.

3. Costa N, Derumeaux H, Rapp T, Garnault V, Ferlicoq L, Gillette S, et al. Methodological considerations in cost of illness studies on Alzheimer disease. Health Econ Rev. 2012;2(1):18. doi: 10.1186/2191-1991-2-18

4. Molinier L, Bauvin E, Combescure C, Castelli C, Rebillard X, Soulie M, et al. Methodological considerations in cost of prostate cancer studies: a systematic review. Value Health 2008;11(5):878-885.

5. Polinder S., Segui-Gomez M., Toet H., Belt E., Sethi D., Racioppi F., et al. Systematic review and quality assessment of economic evaluation studies of injury prevention. Accid Anal Prev. 2012. 45, 211-221.
6. Drummond, M., Sculpher, M., Torrance, G., O'brien, B., Stoddart, G., and Buskens, E. Methods for the economic evaluation of health care programmes. 3rd edition. 2005. Oxford: Oxford University Press.
