# Supplementary material for: In-hospital costs after severe traumatic brain injury: A systematic review and quality assessment
Source: PLoS One. 2019 May 9;14(5):e0216743. doi: 10.1371/journal.pone.0216743 (PMC6508680; doi:10.1371/journal.pone.0216743)
Supplement: S2 Table — * Item scores with double weight. (DOCX) [file pone.0216743.s004.docx]

**S1 Table. Results of the Quality Assessment**

* Item scores with double weight.

| **Quality Assessment Checklist** | | | Reference # | 1 | 2 | 3 | 4 | 5 | 6 | 7 | 8 | 9 | 10 | 11 | 12 | 13 | 14 | 15 | 16 | 17 | 18 | 19 | 20 | 21 | 22 | 23 | 24 | 25 | Score (%) |
| --- | --- | --- | --- | --- | --- | --- | --- | --- | --- | --- | --- | --- | --- | --- | --- | --- | --- | --- | --- | --- | --- | --- | --- | --- | --- | --- | --- | --- | --- |
| Item | # | Study details | |  |  |  |  |  |  |  |  |  |  |  |  |  |  |  |  |  |  |  |  |  |  |  |  |  |  |
| Study details | 1 | Is a description of study objectives and/or study questions provided? | | 1 | 1 | 1 | 1 | 1 | 1 | 1 | 1 | 1 | 1 | 1 | 1 | 1 | 1 | 1 | 1 | 1 | 1 | 1 | 1 | 1 | 1 | 1 | 1 | 1 | 100 |
|  | 2 | Are all relevant aspects of the study setting provided? | | 1 | 1 | 1 | 1 | 1 | 1 | 1 | 1 | 1 | 1 | 1 | 1 | 1 | 1 | 1 | 1 | 1 | 1 | 1 | 1 | 1 | 1 | 1 | 1 | 1 | 100 |
|  | 3 | Is there a statement about the broader context or relevance for health policy or practice decisions? | | 1 | 1 | 1 | 1 | 1 | 1 | 1 | 1 | 1 | 1 | 1 | 1 | 1 | 1 | 1 | 1 | 1 | 1 | 1 | 1 | 1 | 1 | 1 | 1 | 1 | 100 |
|  | Subtotal score (N/3) | | | 3 | 3 | 3 | 3 | 3 | 3 | 3 | 3 | 3 | 3 | 3 | 3 | 3 | 3 | 3 | 3 | 3 | 3 | 3 | 3 | 3 | 3 | 3 | 3 | 3 | 100 |
| Population | 4 | Is there a description of base population and/or subgroups? | | 1 | 1 | 1 | 1 | 1 | 1 | 1 | 1 | 1 | 1 | 1 | 1 | 1 | 1 | 1 | 1 | 1 | 1 | 1 | 1 | 1 | 1 | 1 | 1 | 1 | 100 |
|  | 5 | * Was a clear definition of illness provided? | | 1 | 2 | 2 | 1 | 1 | 1 | 2 | 2 | 1 | 1 | 1 | 2 | 1 | 2 | 2 | 2 | 1 | 2 | 2 | 1 | 1 | 1 | 2 | 2 | 1 | 74 |
|  | 6 | Is there a description of TBI severity? | | 1 | 1 | 1 | 1 | 1 | 1 | 1 | 1 | 1 | 1 | 1 | 1 | 1 | 1 | 1 | 1 | 1 | 1 | 1 | 1 | 1 | 1 | 1 | 1 | 1 | 100 |
|  | Subtotal score (N/4) | | | 3 | 4 | 4 | 3 | 3 | 3 | 4 | 4 | 3 | 3 | 3 | 4 | 3 | 4 | 4 | 4 | 3 | 4 | 4 | 3 | 3 | 3 | 4 | 4 | 3 | 87 |
| Clinical data | 7 | * Were hospital activity data sources carefully described? | | 1 | 2 | 1 | 2 | 2 | 2 | 2 | 2 | 0 | 0 | 1 | 2 | 2 | 2 | 2 | 1 | 2 | 1 | 2 | 2 | 2 | 2 | 2 | 2 | 2 | 82 |
|  | 8 | Were hospital activity data appropriately assessed and mentioned? | | 0.5 | 0.5 | NA | 0.5 | 0.5 | 0.5 | 1 | 1 | 0.5 | 0.5 | 0 | 0.5 | 1 | 1 | 1 | 1 | 1 | 0.5 | 0 | 1 | 1 | NA | 1 | 1 | NA | 70 |
|  | 9 | Was outcome data presented in the study? | | 1 | 1 | 1 | 0 | 1 | 1 | 1 | 1 | 1 | 1 | 0 | 1 | 1 | 1 | 1 | 1 | 0 | 1 | 0 | 1 | 1 | 1 | 1 | 1 | 1 | 84 |
|  | Subtotal score (N/4) | | | 2.5 | 3.5 | 2 | 2.5 | 3.5 | 3.5 | 4 | 4 | 1.5 | 1 | 1.5 | 3.5 | 4 | 4 | 4 | 3 | 3 | 2.5 | 2 | 4 | 4 | 3 | 4 | 4 | 3 | 78 |
| Cost data | 10 | * Were cost data sources carefully described? | | 1 | 2 | 2 | 2 | 2 | 1 | 2 | 2 | 2 | 1 | 2 | 1 | 2 | 2 | 2 | 1 | 2 | 1 | 2 | 2 | 0 | 2 | 2 | 2 | 2 | 84 |
|  | 11 | * Are the reference year and currency provided? | | 1 | 2 | 2 | 2 | 1 | 2 | 2 | 2 | 2 | 1 | 2 | 1 | 2 | 2 | 1 | 1 | 2 | 1 | 2 | 2 | 2 | 2 | 2 | 2 | 2 | 86 |
|  | 12 | * Were unit costs appropriately valued? | | 0 | 0 | 1 | 0 | 1 | 0 | 0 | 0 | 0 | 0 | 0 | 0 | 0 | 0 | 2 | 0 | 0 | 0 | 0 | 0 | 0 | 2 | 1 | 0 | 2 | 18 |
|  | 13 | * Were hospital costs sufficiently disaggregated? | | 0 | 1 | 0 | 2 | 1 | 0 | 0 | 1 | 2 | 0 | 0 | 0 | 0 | 0 | 0 | 0 | 1 | 0 | 0 | 0 | 0 | 0 | 0 | 0 | 2 | 20 |
|  | 14 | * Were the design and methods of costs analysis carefully described | | 0 | 2 | 1 | 2 | 0 | 0 | 1 | 2 | 2 | 0 | 1 | 0 | 1 | 2 | 2 | 0 | 2 | 0 | 0 | 2 | 0 | 2 | 2 | 2 | 2 | 56 |
|  | 15 | Were the major assumptions tested in a sensitivity analysis | | 0 | 0 | 1 | 0 | 0 | 0 | 0 | 1 | 0 | 0 | 0 | 0 | 0 | 0 | 0 | 0 | 0 | 0 | 0 | 0 | 0 | 1 | 0 | 0 | 1 | 16 |
|  | Subtotal score (N/11) | | | 2 | 7 | 7 | 8 | 5 | 3 | 5 | 8 | 8 | 5 | 2 | 2 | 5 | 6 | 7 | 2 | 7 | 2 | 4 | 6 | 2 | 9 | 7 | 6 | 11 | 49 |
| Methodology | 16 | Were study limitations carefully described using study objective, methods and results? | | 0 | 1 | 1 | 1 | 1 | 1 | 1 | 1 | 1 | 1 | 1 | 1 | 1 | 1 | 1 | 1 | 1 | 0.5 | 1 | 0 | 1 | 1 | 1 | 1 | 1 | 90 |
|  | 17 | Was there a comparison of the findings with current knowledge? | | 1 | 1 | 1 | 0 | 0.5 | 1 | 0 | 0.5 | 0.5 | 1 | 1 | 1 | 1 | 1 | 1 | 1 | 1 | 0.5 | 1 | 0.5 | 0.5 | 1 | 1 | 1 | 1 | 80 |
|  | 18 | Was the presentation of study results consistent with the methodology of the study? | | 1 | 1 | 1 | 1 | 1 | 1 | 1 | 1 | 1 | 1 | 1 | 1 | 1 | 1 | 1 | 1 | 1 | 1 | 1 | 1 | 1 | 1 | 1 | 1 | 1 | 100 |
|  | 19 | Is the source of funding and are possible conflicts of interest mentioned? | | 0 | 1 | 0.5 | 0.5 | 0 | 0 | 0.5 | 1 | 0.5 | 0.5 | 1 | 1 | 0.5 | 0.5 | 1 | 0 | 1 | 1 | 0 | 0 | 0 | 1 | 1 | 1 | 1 | 54 |
|  | Subtotal score (N/4) | | | 2 | 4 | 3.5 | 2.5 | 2.5 | 3 | 2.5 | 3.5 | 3 | 4 | 3.5 | 4 | 3.5 | 3.5 | 4 | 3 | 4 | 3 | 3 | 1.5 | 2.5 | 4 | 4 | 4 | 4 | 82 |
| Total score (%) | | | | 48 | 83 | 78 | 73 | 65 | 60 | 71 | 87 | 71 | 62 | 50 | 63 | 72 | 79 | 86 | 58 | 77 | 56 | 62 | 67 | 56 | 88 | 85 | 81 | 96 |  |
